# Polydnaviral Ankyrin Proteins Aid Parasitic Wasp Survival by Coordinate and Selective Inhibition of Hematopoietic and Immune NF-kappa B Signaling in Insect Hosts

**DOI:** 10.1371/journal.ppat.1003580

**Published:** 2013-08-29

**Authors:** Gwenaelle Gueguen, Marta E. Kalamarz, Johnny Ramroop, Jeffrey Uribe, Shubha Govind

**Affiliations:** 1 Biology Department, The City College of the City University of New York, New York, New York, United States of America; 2 The Graduate Center of the City University of New York, New York, New York, United States of America; Stanford University, United States of America

## Abstract

Polydnaviruses are mutualists of their parasitoid wasps and express genes in immune cells of their Lepidopteran hosts. Polydnaviral genomes carry multiple copies of viral *ankyrins* or *vankyrins*. Vankyrin proteins are homologous to IκB proteins, but lack sequences for regulated degradation. We tested if Ichnoviral Vankyrins differentially impede Toll-NF-κB-dependent hematopoietic and immune signaling in a heterologous *in vivo Drosophila*, system. We first show that hematopoiesis and the cellular encapsulation response against parasitoid wasps are tightly-linked via NF-κB signaling. The niche, which neighbors the larval hematopoietic progenitors, responds to parasite infection. *Drosophila* NF-κB proteins are expressed in the niche, and non cell-autonomously influence fate choice in basal and parasite-activated hematopoiesis. These effects are blocked by the Vankyrin I^2^-vank-3, but not by P-vank-1, as is the expression of a NF-κB target transgene. I^2^-vank-3 and P-vank-1 differentially obstruct cellular and humoral inflammation. Additionally, their maternal expression weakens ventral embryonic patterning. We propose that selective perturbation of NF-κB-IκB interactions in natural hosts of parasitic wasps negatively impacts the outcome of hematopoietic and immune signaling and this immune deficit contributes to parasite survival and species success in nature.

## Author Summary

Parasitoid wasps are insects whose development takes place within the body of other insects. To survive, wasp larvae must overcome the immune defenses of their hosts. How parasitic wasps overcome host immunity is not fully understood even though we know that different strategies using venoms, virus-like particles, or viruses are involved. A unique class of viruses, called polydnaviruses is found in two families of wasps that comprise more than 20,000 wasp species. The genomes of polydnaviruses encode proteins with ankyrin repeats. Ankyrin repeats are also found in Cactus, the inhibitor protein of NF-κB signaling in *Drosophila*. Viral ankyrin proteins, or Vankyrins, however, lack the amino acid sequences necessary for turnover found in Cactus and mammalian IκB family members. We show that Vankyrins produced by polydnaviruses of a parasitic wasp that attacks caterpillars of many common agricultural pests can block NF-κB signaling in fruit fly larvae. This inhibition supports parasite success. Our work highlights the crucial role of NF-κB signaling across insect taxa in insect-insect and insect-virus interactions. Studies of polydnaviral ankyrin proteins in *Drosophila* reveal that immune-suppressive viruses may block both cellular and humoral immunity in insects to win the biological ‘arms race’.

## Introduction

Parasitic wasps develop within their insect hosts as they devour host bodies. Wasp oviposition in *Drosophila* larvae simultaneously activates humoral and cellular immune reactions. In a systemic acute inflammatory reaction, humoral antimicrobial secretions and cytokines from the fat body synergize with hematopoietic proliferation and differentiation, to encapsulate the wasp egg and protect host larvae [1], [2], [3]. Immune response against wasp eggs alters hematopoietic development in the larval lymph gland and in the hemolymph [4], [5], [6]. Genetic and molecular analysis of wasp-infected *Drosophila* hosts has revealed the fundamental role for the Toll-NF-κB pathway in both humoral and cellular immunity [2], [3], [7]. Toll signaling is also essential for basal hematopoiesis in the lymph gland [8], although the precise functions of the Toll effector proteins, the NF-κB family transcription factors Dorsal (dl) and Dorsal-related immunity factor (Dif), in either basal or activated hematopoiesis are not understood.

The nuclear translocation and functions of Dorsal and Dif are inhibited by their interactions with Cactus, the cognate IκB inhibitor [9], [10]. The direct physical interaction with NF-κB proteins depends on several ankyrin repeats in the IκB protein sequences [11], [12]. A Toll-dependent degradation signal is interpreted by the N-terminal regulatory domain of Cactus [13]. Indeed, chronic inflammatory defects wrought by excessive Toll activation are ameliorated by a mutant Cactus without the N-terminal domain responsible for signal-dependent degradation [14]. Interestingly, ankyrin repeat sequence motifs, homologous to Cactus, are found in the genomes of all sequenced polydnaviruses [15], [16], [17]. However, whether closely-related members of this large family of insect viral proteins support parasite development by redundantly or differentially blocking NF-κB signaling in host hematopoiesis and immunity, and mechanisms underlying such differences, is not known.

Double-stranded DNA-carrying mutualistic and pathogenic polydnaviruses (PDVs) fall into the evolutionarily distinct bracovirus (BV) and ichnovirus (IV) genera that are associated with an estimated 20,000 species of parasitic wasp families Braconidae and Ichneumonidae, respectively [18]. Each wasp species has co-evolved with a unique, vertically-transmitted PDV [19], [20] that they introduce into their Lepidopteran larval hosts upon oviposition. The polydnaviral particles express their gene products in infected tissues to ensure wasp success [18].

The viral ankyrin (*vankyrin*) gene family is common to both BV and IV genomes; each genome carries several members [21]. The *Campoletis sonorensis* IV (CsIV) genome contains seven copies of *vankyrin* genes, with 47% to 83% amino acid sequence identity. Because Vankyrins lack the N- and C-terminal regulatory domains of cellular IκBs, it was suggested that these proteins effectively inhibit NF-κB signaling in parasitized insects [16], [22].

In this study, we first defined new functions of Dif and Dorsal in basal and activated hematopoiesis. We then tested the inhibitory functions of two of the seven *vankyrin* genes of the *Campoletis sonorensis* Ichnovirus, CsIV-P-vank-1 (P1) and CsIV-I^2^-vank-3 (I3). These Vankyrins (1) are the most similar to each other, with 83% amino acid sequence identity; (2) are derived from different DNA segments (P and I) of the multipartite CsIV genome; (3) share only four of the six 33-amino acid ankyrin repeats of Cactus; (4) possess a putative functional zinc-binding motif in their N-termini not present in other PDV ankyrins; (5) and are also among the most expressed in Lepidoptera immune tissues after parasitization. We reasoned that a Vankyrin-based immune-suppressive strategy between BVs and IVs reflects the broad functional conservation of NF-κB-dependent immune responses in insects and an intrinsic ability of Vankyrins to dominantly interfere with Toll-NF-κB signaling in a context-independent manner.

In a novel application of *Drosophila* to examine insect-insect and insect-virus interactions, we tested if P1 and I3 might differentially block hematopoietic and immune signaling in *Drosophila* models of acute (*Leptopilina* spp. wasp egg encapsulation) and chronic (ectopic NF-κB signaling) inflammation [2]. We also examined their ability to temper the maternal NF-κB pathway essential for embryonic dorsal/ventral (d/v) axis formation. We report specific and dose-dependent inhibition of NF-κB signaling in hematopoiesis, innate immunity, and embryonic patterning by P1 and I3. These results suggest that NF-κB signaling is pervasive across taxa and offer rational means for its selective inhibition by viral-ankyrin proteins.

## Results

### The lymph gland niche is sensitive to wasp infection and activates NF-κB signaling

**Figure 1:**
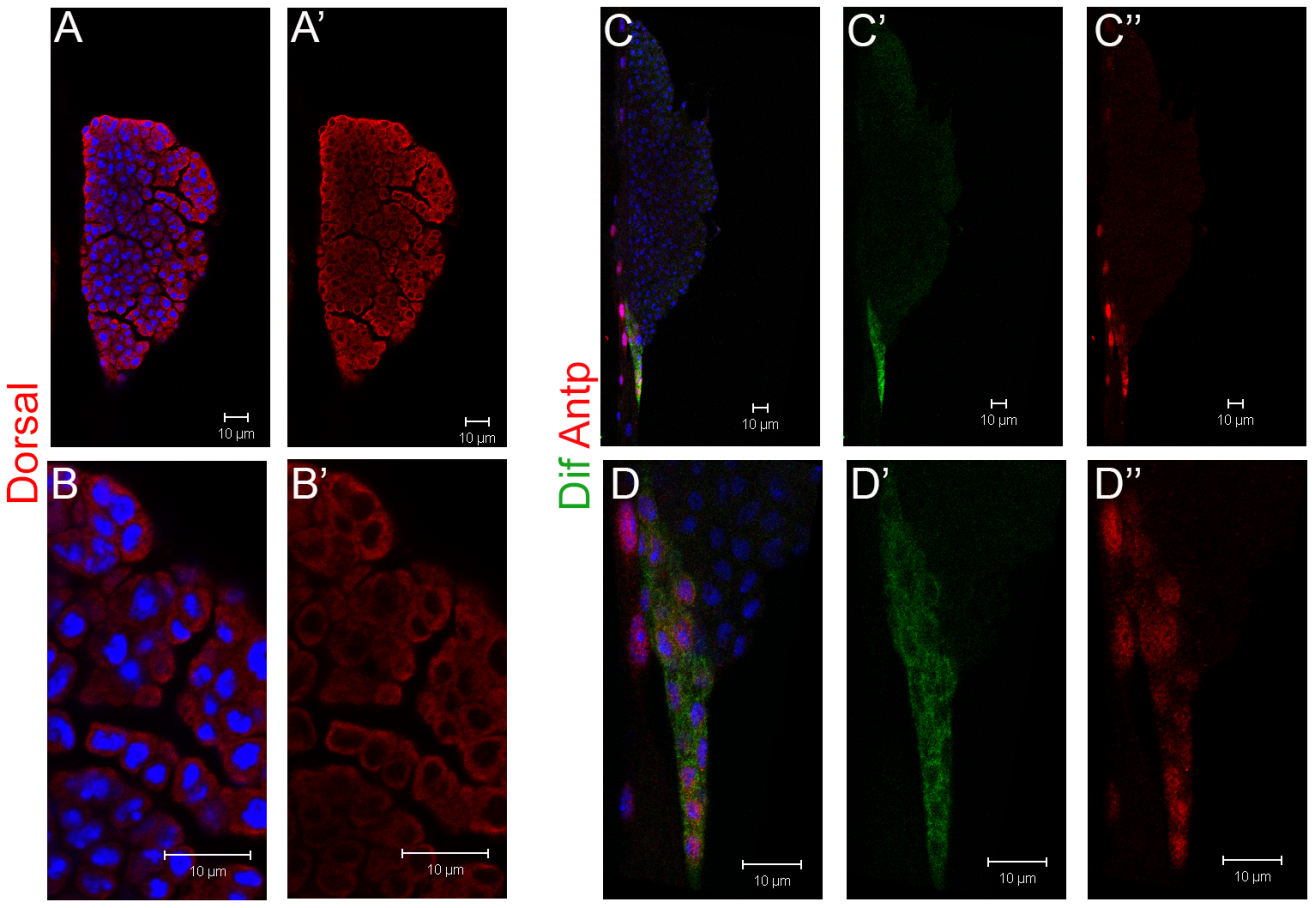
Dif and Dorsal localization in third instar lymph gland. **A–B′**. Dorsal is found throughout the anterior lobes of a third instar control (*Ubc9^−^/CyO y^+^*) lymph gland. **C–D″**. Dif signal is high in Antp-positive niche cells of the control (*Ubc9^−^/CyO y^+^*) lymph gland. Staining signal was not detected in mutants lacking either gene (not shown). Bars indicate 10 µm.

Sumoylation-deficient animals exhibit ectopic NF-κB signaling which correlates with persistent high levels of an active ligand for the Toll receptor (Spatzle), and low levels of Cactus protein in immune cells. These changes contribute to continuous hematopoietic overproliferation and chronic inflammation [2], [23]. Loss of the *Dif* and *dl* loci (or of *dl* alone) suppresses chronic inflammation and aberrant hematopoiesis of *Ubc9* mutants [14]. Wasp infection of *Drosophila* larvae activates NF-κB functions [2], [3] and also alters hematopoietic development in the lymph gland [5], [7], [24]. These results suggested that Dorsal and Dif likely control normal hematopoiesis in the lymph gland and their activity may be subject to immune suppression by parasitic wasps. The lymph gland is a lobed organ. Anterior lobes of the third instar larva are most developed and harbor a quiescent medullary zone (MZ) with relatively undifferentiated cells, maintained by the niche or posterior signaling center, while the peripheral cortical zone (CZ) contains cells at various developmental stages, including mature immune cells [25]. The niche is specified by the homeobox transcription factor Antennapedia (Antp) [26]. The phagocytic plasmatocytes make up the majority of mature cells, while crystal cells carry pro-phenol-oxidase crystals for melanization. Specialized large adhesive cells, lamellocytes, differentiate when Toll-NF-κB signaling is hyperactive or upon wasp infection [1]. In antibody staining experiments, we found Dorsal expression throughout the lymph gland lobes with somewhat higher signal in the CZ (Fig. 1A–B′). In contrast, Dif expression is high in the Antennapedia (Antp)-expressing niche cells. Dif signal is lower and variable in the MZ/CZ regions (Fig. 1C–D″).

**Figure 2:**
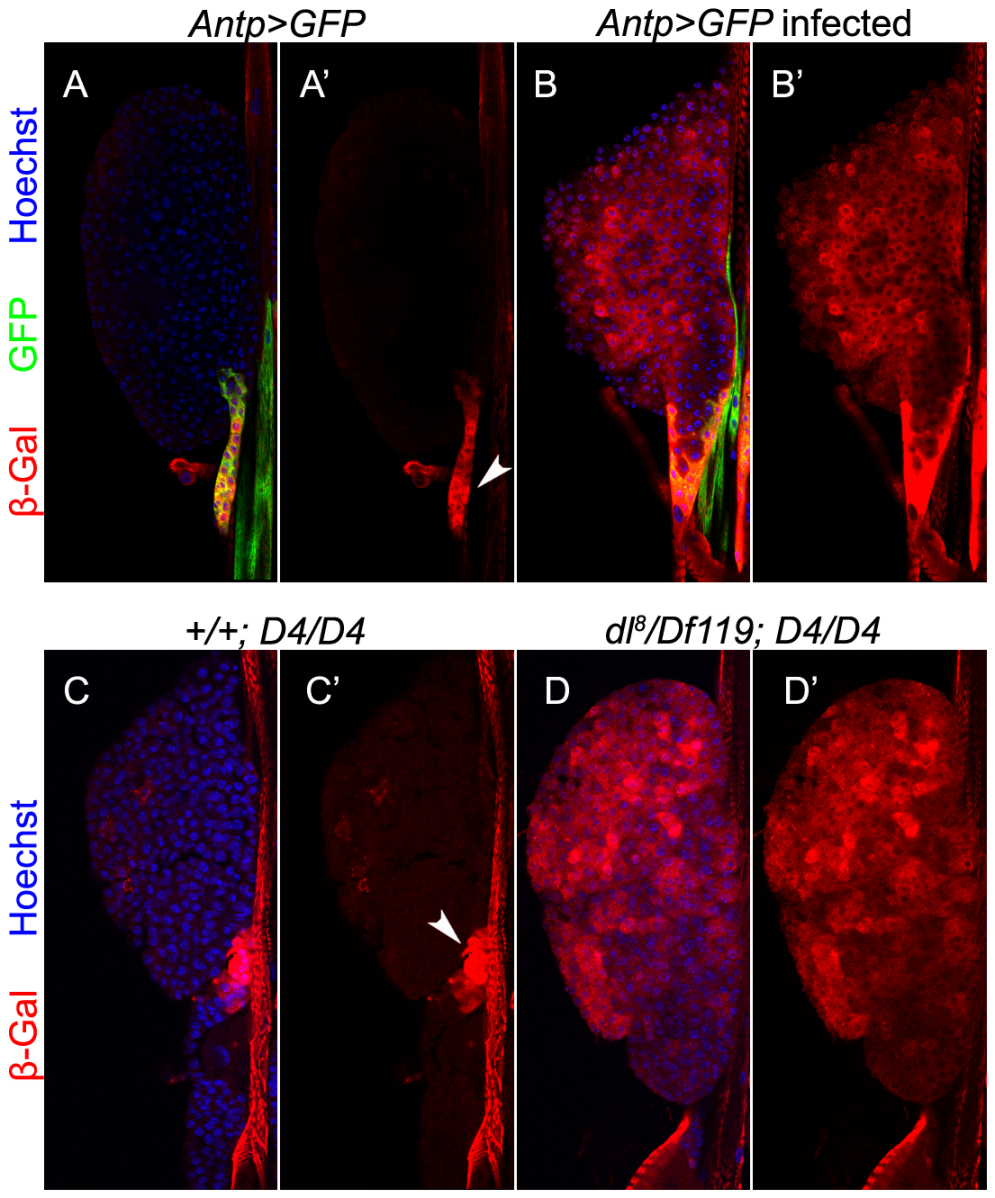
Effect of wasp infection on *D4-lacZ* expression. **A–A′**. Uninfected *Antp>GFP* animals. *D4-lacZ* is expressed mostly in the niche (A′, arrowhead) where it colocalizes with *Antp>GFP* (A, yellow). **B–B′**. *L. boulardi* infection triggers four-fold increase in the expression of *D4-lacZ* (23.06±4.22 versus 89.65±41.4; t = −6.37, df = 14.7, p<0.001; N = 4 glands for uninfected and 8 for infected). *D4-lacZ* is also activated in cells of the anterior lobes. **C–D′**. Niche expression of *D4-lacZ* is abolished in glands lacking a functional *dl* gene (D, D′), but is observed in controls (**C–C′**). The reporter is expressed ectopically in the mutant but not control lobe cortex.

We utilized a *D4-lacZ* reporter, which contains four Dorsal/Dif binding sites [27]. In uninfected larvae, *D4-lacZ* expression (Fig. 2A–A′) co-localizes with *Antp>GFP* expression. This basal *D4-lacZ* expression suggests high Dorsal/Dif transcriptional activity in the niche even in uninfected animals. Upon *L. boulardi* infection, *D4-lacZ* expression is four times higher in infected compared to uninfected larvae (compare Fig. 2B′ to Fig. 2A′). Additionally, numerous cells of the anterior lobes are also positive for anti-β-galactosidase staining (Fig. 2B–B′). The basal *D4-lacZ* expression in the niche is not observed in glands from *dl^8^/Df119* animals (compare Fig. 2C to Fig. 2D), although, surprisingly, these mutant glands express the *D4-lacZ* reporter in many cortical cells (Fig. 2C–D′). Thus, it appears that (a) consistent with NF-κB function in anti-wasp response, the *D4-lacZ* reporter is sensitive to and is differentially activated (in distinct cell populations) by parasitization; (b) transcriptional activity of Dorsal in the niche is essential for *D4-lacZ* expression; and (c) Dorsal possibly represses transcription of gene targets in the lobe cortex.

### Dif and Dorsal regulate basal hematopoiesis in the niche

**Figure 3:**
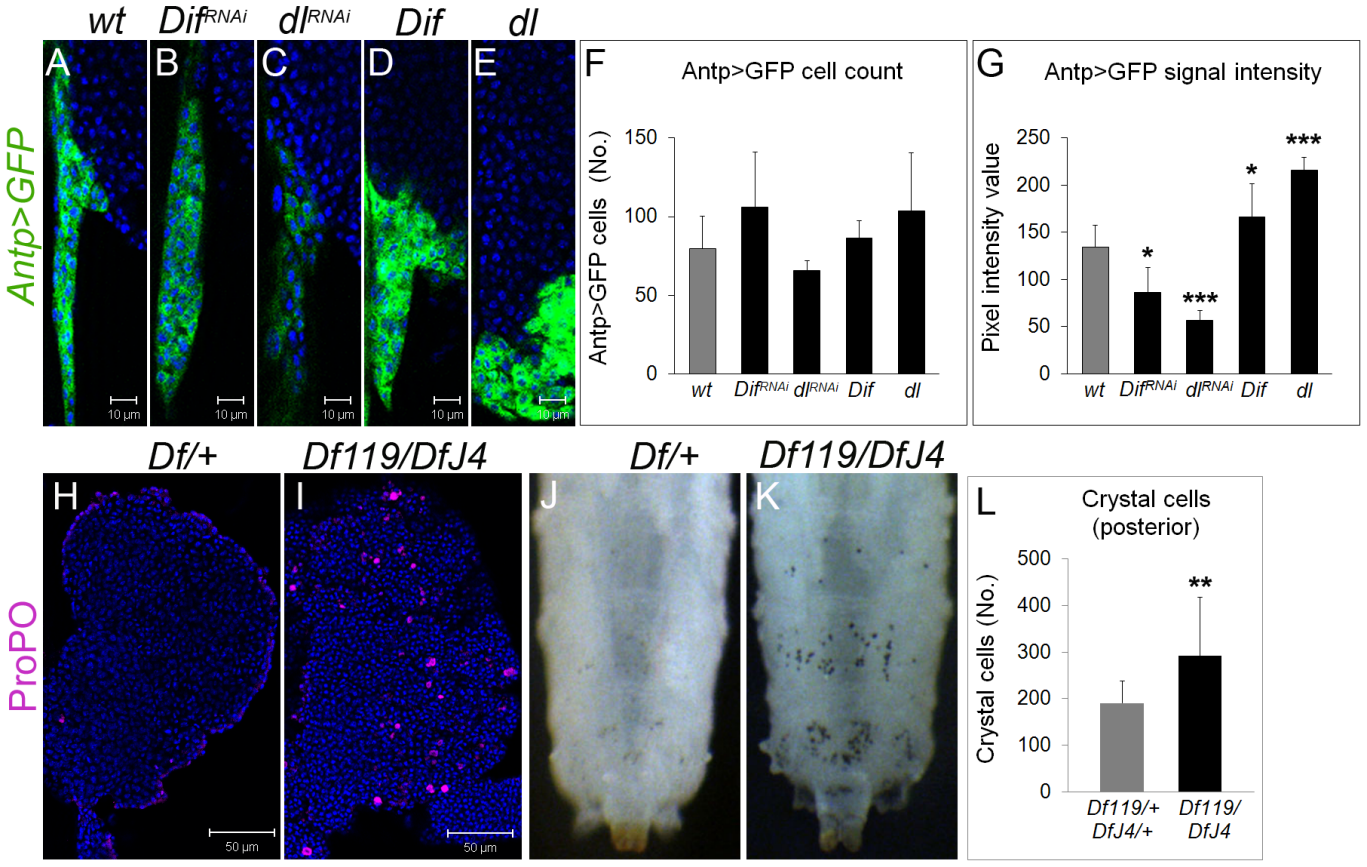
Effects of Dif and Dorsal on *Antp>GFP* expression and on crystal cell number. (**A–E**) Antp>GFP expression in third instar lymph glands. **A**. Control. **B**. Knockdown of Dif (*Dif^RNAi^*), or **C**. Dorsal (*dl^RNAi^*). Overexpression of **D**. Dif (*Dif*), or **E**. Dorsal (*dl*). **F**. Manipulation of Dif or Dorsal levels does not affect the number of *Antp>GFP*-positive cells (t = −1.45, df = 6.5, p = 0.19 for *Antp>Dif^RNAi^*, t = 1.45, df = 4.7, p = 0.21 for *Antp>dl^RNAi^*, t = −0.62, df = 6.2, p = 0.55 for *Antp>Dif* and t = −1.29, df = 6.3, p = 0.24 for *Antp>dl*). **G**. Quantification of *Antp>GFP* expression. The intensity of the GFP signal is reduced in *Antp>Dif^RNAi^* (t = 3.4, df = 7.8, p = 0.01) and *Antp>dl^RNAi^* (t = 7.8, df = 5.6, p<0.001) and increased in *Antp>Dif* (t = −2.4, df = 8, p = 0.04) and *Antp>dl* (134.6 versus 216.2 - t = −8.2, df = 5.4, p<0.001), compared to controls, N = 5 animals for each genotype. **H–I**. Anterior lobes of animals **H**. heterozygous for the deficiency lacking *Dif* and *dl* (*Df(2L)TW119/+* or *Df(2L)J4/+*), and **I**. *Dif/dl* mutants (*Df(2L)TW119/Df(2L)J4*) stained for ProPO2 (magenta, crystal cells). (**J–K**) Crystal cells (black spots) of third instar, **J**. heterozygous control, and **K**. *Df(2L)TW119/Df(2L)J4* mutant, visualized by incubation at 70°C. **L**. The average number of sessile crystal cells in the three posterior segments is significantly increased in *Dif^−^ dl^−^* mutants (t = −3.4, df = 25.9, p = 0.002. N = 20 for heterozygotes; N = 21 for *Dif^−^ dl^−^* mutants). Bars indicate standard deviation. Stars indicate statistical significance relative to controls (* for 0.05<p<0.01, ** for 0.01<p<0.001 and *** for p<0.001).

To test their individual effects on the niche and on hematopoiesis, we modulated NF-κB levels in the niche. (1) RNAi knockdown with either *Antp>GFP, Dif^RNAi^* or *Antp>GFP, dl^RNAi^* did not yield a significant difference in the number of GFP-positive cells (Fig. 3A–C, F), although, unexpectedly, the intensity of the *Antp>GFP* signal in cells with RNAi was significantly reduced (Fig. 3A–C, G). Conversely, ectopic expression of Dif or Dorsal in the niche increased *Antp>GFP* expression (Fig. 3D–E, G). (2) Overexpression of either wild type protein (*Antp>dl* or *Antp>Dif*) also showed supernumerary lamellocytes in the lymph gland lobes (Fig. S1G, J–K). (3) We found no significant difference in the number of crystal cells in *Antp>GFP, Dif^RNAi^* or *Antp>GFP, dl^RNAi^* glands, slight increase in *Antp>GFP, Dif* but no change in *Antp>GFP, dl* (Fig. S1A–F). However, *Df(2L)119/Df(2L)J4* mutants lacking both NF-κB proteins show significantly more crystal cells in the lymph gland and in the sessile compartment, compared to heterozygous controls (Fig. 3H–L), indicating inhibitory and redundant NF-κB roles in crystal cell development. Anti-Antp staining of lobes from *Df(2L)119/Df(2L)J4* glands revealed that the niche is specified in the absence of Dif and Dorsal and the Antp protein expression levels appears comparable to those in heterozygous controls (data not shown). Together, these results suggest that Dorsal and Dif can modulate *Antp-Gal4* transgene expression in the niche and are required non cell-autonomously for the proportional development of crystal cell and lamellocytic lineages.

We next made flp-out clones marked with GFP alone (control) or additionally expressing the fusion protein GFP-Dorsal to test for non cell-autonomous requirement. Consistent with observations above, glands with GFP-Dorsal clones showed a reduction in crystal cells (Fig. S2A–C) and an increase in lamellocytes (Fig. S2D–F), relative to glands with control clones. In both cases, mature cells appeared GFP-negative and outside the clone boundary (Fig. S2B, E). This observation supports a non cell-autonomous requirement for Dorsal/NF-κB in the development of these lineages.

### Expression and effects of P1 and I3 on niche and basal/activated hematopoiesis

**Figure 4:**
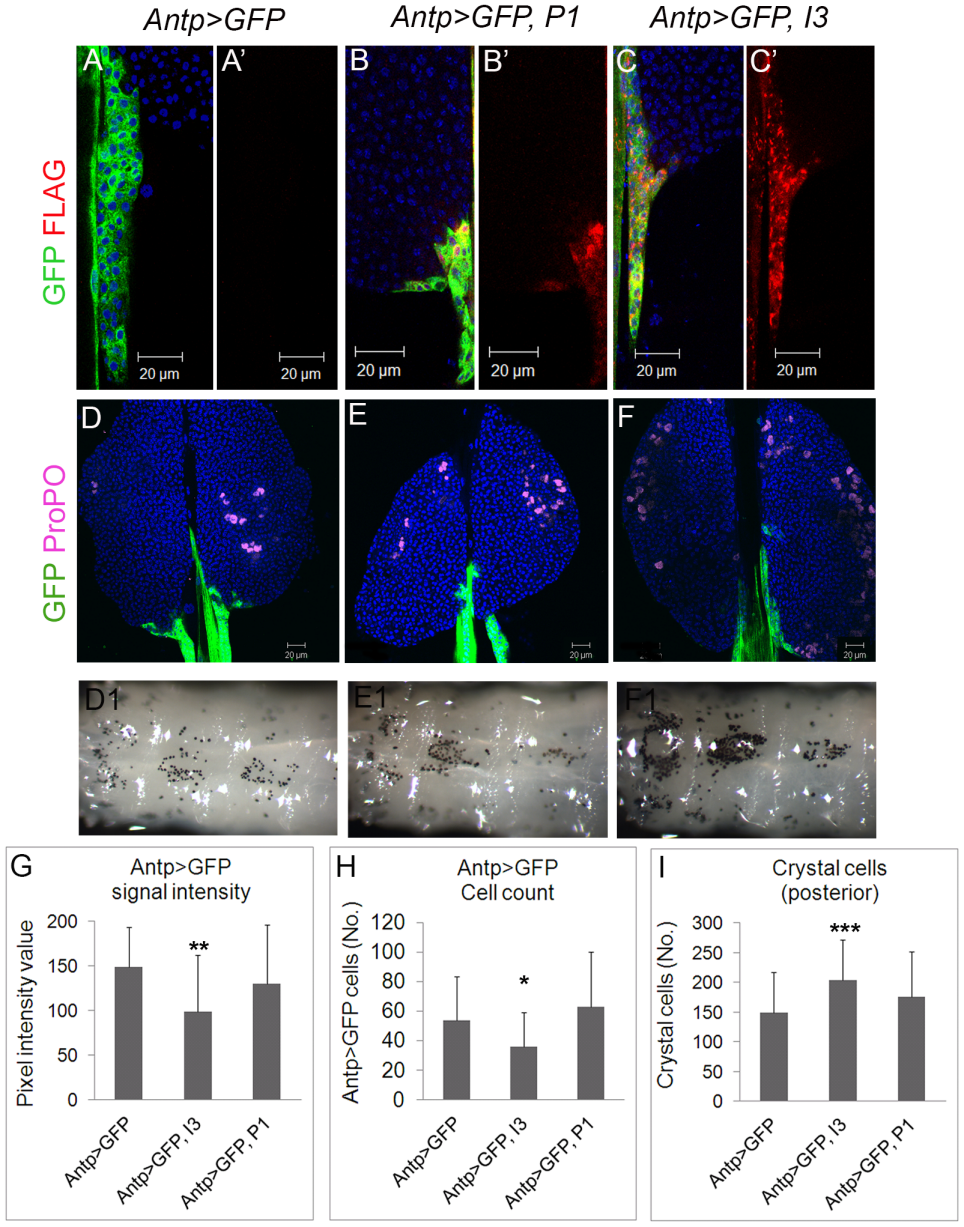
Vankyrin localization and their effects on niche properties and on crystal cell development. **A–A′**. Niche without any Vankyrin expression stained with anti-FLAG antibody. **B–B′**. Antp-Gal4 simultaneously drives the expression of GFP and P1 from their respective UAS sequences. P1 localizes to nuclei and in the cytoplasm (yellow) where it is relatively uniformly distributed. **C–C′**. Antp-Gal4 simultaneously drives the expression of GFP and I3 from their respective UAS sequences. I3 is expressed mostly in the cytoplasm; it colocalizes with *Antp>GFP* expression in some cells (yellow), and its distribution is speckled. **D–F1**. Effect of Vankyrins on crystal cells development. **D–F**. Crystal cells in the anterior lobes of the lymph gland. Crystal cell number is not significantly different from the control when either Vankyrin is expressed. **D1–F1**. However, their number is increased in the three posterior larval segments when I3 is expressed (for quantification, see Panel I.) **G**. Expression of I3 reduces the number of *Antp>GFP*-positive cells in the niche compared to controls (W = 438, p = 0.03 while P1 does not (W = 229, p = 0.09). N = 16 animals for control; N = 10 animals for I3 and P1-expressing glands. Cell counts represent an average per niche. **H**. Expression of I3 decreases the intensity (measurement done on more than 15 cells – see Methods) of *Antp>GFP* signal. Pixel intensity is reduced in *Antp>GFP, I3* (t = 3.3, df = 30.9, p = 0.002) but not in *Antp>GFP, P1* (t = 1.9, df = 47.6, p = 0.07) compared to controls (N = 15 animals for *Antp>GFP* controls, N = 9 animals for I3 expressing animals and N = 10 for P1 expressing animals). **I**. Quantification of crystal cell changes in panels E1 and F1, relative to D1. Crystal cell number in the three posterior larval segments is increased by *Antp>I3* (t = −3.7, df = 65, p<0.001; control, N = 33; I3 expressing animals, N = 34) but not with *Antp>P1* (t = −1.7, df = 59.197, p = 0.09 - P1 expressing animals, N = 29). Scale bars represent 20 µm. Bars indicate standard deviation. Stars indicate conditions that are different from controls (* for 0.05<p<0.01, ** for 0.01<p<0.001 and *** for p<0.001).

Viral protein, P1 or I3, was co-expressed with GFP using the Antp-Gal4 driver. Both P1 and I3 are not only cytoplasmic (Fig. 4A, B, C; yellow), but also nuclear in cells of the niche (Fig. 4B, C; purple). While P1 is more uniformly distributed (Fig. 4B′), I3 distribution is speckled (Fig. 4C′).

Antp>P1 expression had no detectable effect on crystal cell numbers (Fig. 4E–E1, I), on the number of cells in the lymph gland niche (Fig. 4H), or on the intensity of *Antp>GFP* signal (Fig. 4G). However, like *Antp>Dif^RNAi^* or *Antp>dl^RNAi^*, Antp>I3 reduces the intensity of Antp>GFP (Fig. 4G), and similar to *Dif^−^ dl^−^* mutants, Antp>I3 increases the number of circulating/sessile crystal cells in the posterior larval segments (Fig. 4F1, I). Additionally, its expression reduces the niche cell count (Fig. 4H).

**Figure 5:**
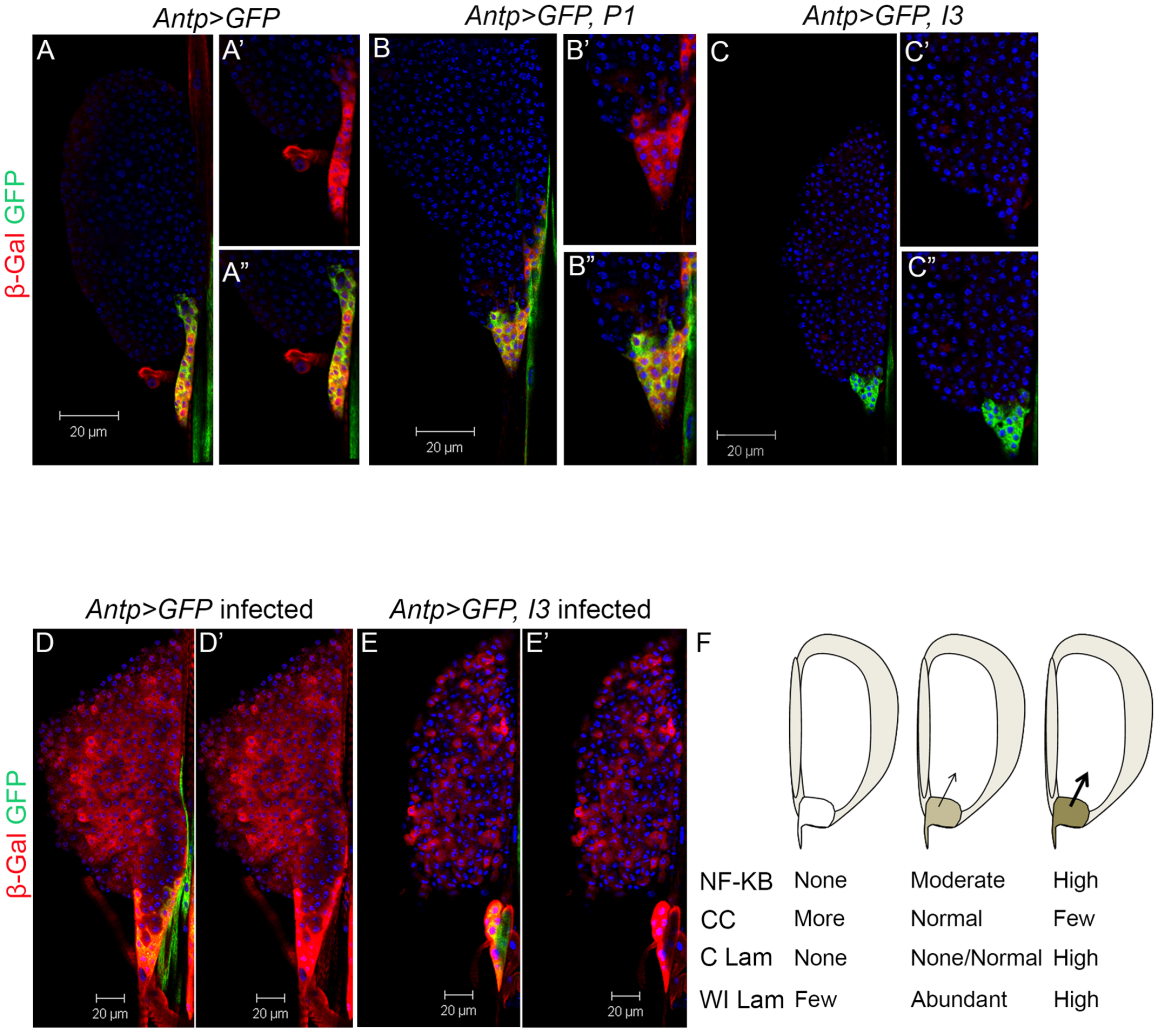
Effect of Vankyrins on *D4-lacZ* expression. **A–A″**. In uninfected *Antp>GFP* animals, *D4-lacZ* reporter is expressed strongly in the niche where it overlaps with *Antp>GFP* (A′–A″ – same image as in Fig. 2A–A″) and is sometimes found in a few cells in the anterior lobes. Expression of the *D4-lacZ* reporter is not changed in the niche when P1 is expressed (**B–B″**) but is clearly reduced by the expression of I3 (**C–C″**) compared to controls. **D–D′**. Same image as in Fig. 2B–B″. Infection strongly induces *D4-lacZ* expression in the niche and cells of the anterior lobes (compare D with A). **E–E′**. Expression of I3 in infected animals limits the induction of *D4-lacZ* in cells of the anterior lobes (compare intensity of β-Gal staining in cells of the anterior lobes in E′ *versus* D′ - t = 5.4, df = 27.1, p<0.001; N = 8 for *Antp>GFP* animals and N = 12 for *Antp>GFP, I3* animals). **F**. Schematic linking NF-κB activity in the niche to choice of cell fate. Wild type control lymph glands (middle) with moderate NF-κB activity develop the correct proportion of crystal cells (CC), possess few if any constitutive lamellocytes (C, Lam), and abundant wasp-induced lamellocytes (WI Lam). Lack of NF-κB activity (left) shifts hematopoiesis in favor of crystal cells, while constitutive lamellocytes are absent and only a few wasp-induced lamellocytes are specified. High NF-κB (right; achieved either by infection or by genetic activation) shifts hematopoiesis in favor of lamellocytes and discourages crystal cell production.

Consistent with the interpretation that I3 may be able to block Dorsal/Dif function, we found, using the *D4-lacZ* reporter, that *Antp>GFP, I3* (but not P1) reduces basal levels of β-galactosidase expression in the niche (Fig. 5A–C″). Moreover, wasp infection-induced boost in *D4-lacZ* expression in the lobes is also significantly inhibited by I3 with reduced β-galactosidase signal intensity (Fig. 5D–F).

### Localization of Vankyrins in circulating blood cells in relation to GFP-Dorsal

**Figure 6:**
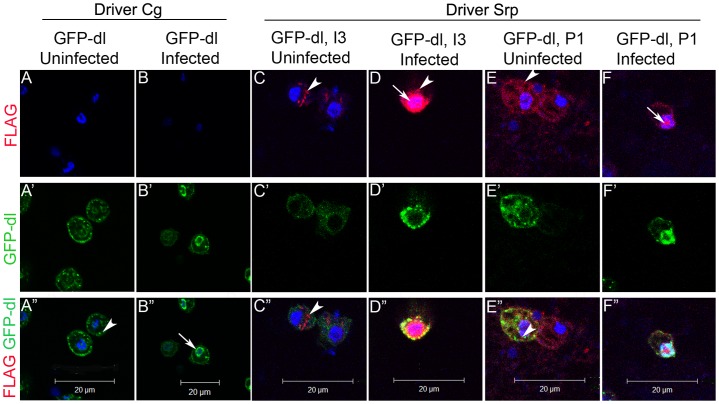
Vankyrins and GFP-Dorsal localization in blood cells. **A–A″**. Uninfected *Cg>GFP-Dorsal* shows speckled distribution (arrowhead). **B–B″**. Infected *Cg>GFP-Dorsal*. Infection relocalizes some GFP-Dorsal to nucleus (arrow). **C–C″**. Uninfected *Srp>GFP, I3*. Both I3 (C, arrowhead) and GFP-Dorsal (C′) are mostly cytoplasmic in blood cells of uninfected animals and do not show much co-localization. **D–D″** Infected *Srp>GFP, I3*. In infected animals, I3 is strongly nuclear (D, arrow) but most of the GFP-Dorsal colocalizes with the remaining cytoplasmic I3 (D″ – yellow). **E–E″**. P1 is also mostly cytoplasmic in cells from uninfected animals (E, E″, arrowhead). **F–F″**. Upon infection, both GFP-Dorsal and P1 colocalize in the nucleus (F″, white). All images are presented at the same magnification and scale bars represent 20 µm. *Cg>GFP-dl, I3* cells from uninfected animals show cytoplasmic localization of both proteins similar to *Srp>GFP-dl I3* (data not shown).

To determine if Vankyrins might co-localize with Dorsal and whether their subcellular localization in relation to Dorsal changes upon wasp-infection, we examined their presence in GFP-Dorsal-expressing blood cells. The GFP-Dorsal fusion protein, when expressed alone (Cg>GFP-Dorsal) is punctuate in the cytoplasm of uninfected larval blood cells and some fusion protein translocates to the nucleus upon wasp infection (Fig. 6A–B″) [2]. I3 distribution in uninfected cells (*Srp>GFP-Dorsal; Srp>I3*) is also cytoplasmic and punctuate (Fig. 6C–C″ - arrowhead). P1 distribution (*Srp>GFP-Dorsal; Srp>P1*) in these blood cells is also largely cytoplasmic and punctuate even though more evenly distributed than I3 (Fig. 6E–E″ - arrowhead). In control cells, both I3 and P1 signals show little to no overlap with the GFP-Dorsal signal (Fig. 6C″, E″). Upon wasp infection, both GFP-Dorsal and Vankyrin signals in blood cells are higher compared to cells from uninfected animals and their distribution is variable. While the I3 signal is intensely nuclear (Fig. 6D - arrow), there is strong and clear co-localization of some I3 with GFP-Dorsal in vesicular pattern in the cytoplasm (Fig. 6D″ - yellow). Interestingly, GFP-Dorsal levels remain relatively low in the nuclei of most blood cells from wasp-challenged I3-expressing animals (Fig. 6D′). In contrast, some of the P1 signal co-localizes with nuclear GFP-Dorsal (Fig. 6F″ - white). These results suggest that, unlike their behavior in cultured Lepidopteran cells (where lipopolysaccharide or laminarin exposure alters localization from nucleus to cytoplasm [28]), in *Drosophila* larval blood cells, both I3 and P1 proteins respond to wasp infection by relocalizing from the cytoplasm to the nucleus. These results suggest that Vankyrins may block signaling by interaction with NF-κB proteins in different subcellular compartments.

### Vankyrins inhibit wasp-induced encapsulation and Toll^10b^-induced tumorogenesis

The circulating blood cells constitute a separate hematopoietic compartment and are derived from embryonic hemocytes [29]. These cells contribute to the cellular immune encapsulation response [30]. To examine if either P1 or I3 affect larval circulating blood cell count (also referred to as circulating hemocyte concentration, or CHC [7]) or *Cg>GFP* expression, we monitored the CHC and GFP expression in *Cg>GFP*, *Cg>GFP, P1*, and *Cg>GFP, I3* larvae and found no significant difference (Fig. S3A). In addition, there was no detectable effect of P1 or I3 on zygotic development or viability (data not shown).

**Figure 7:**
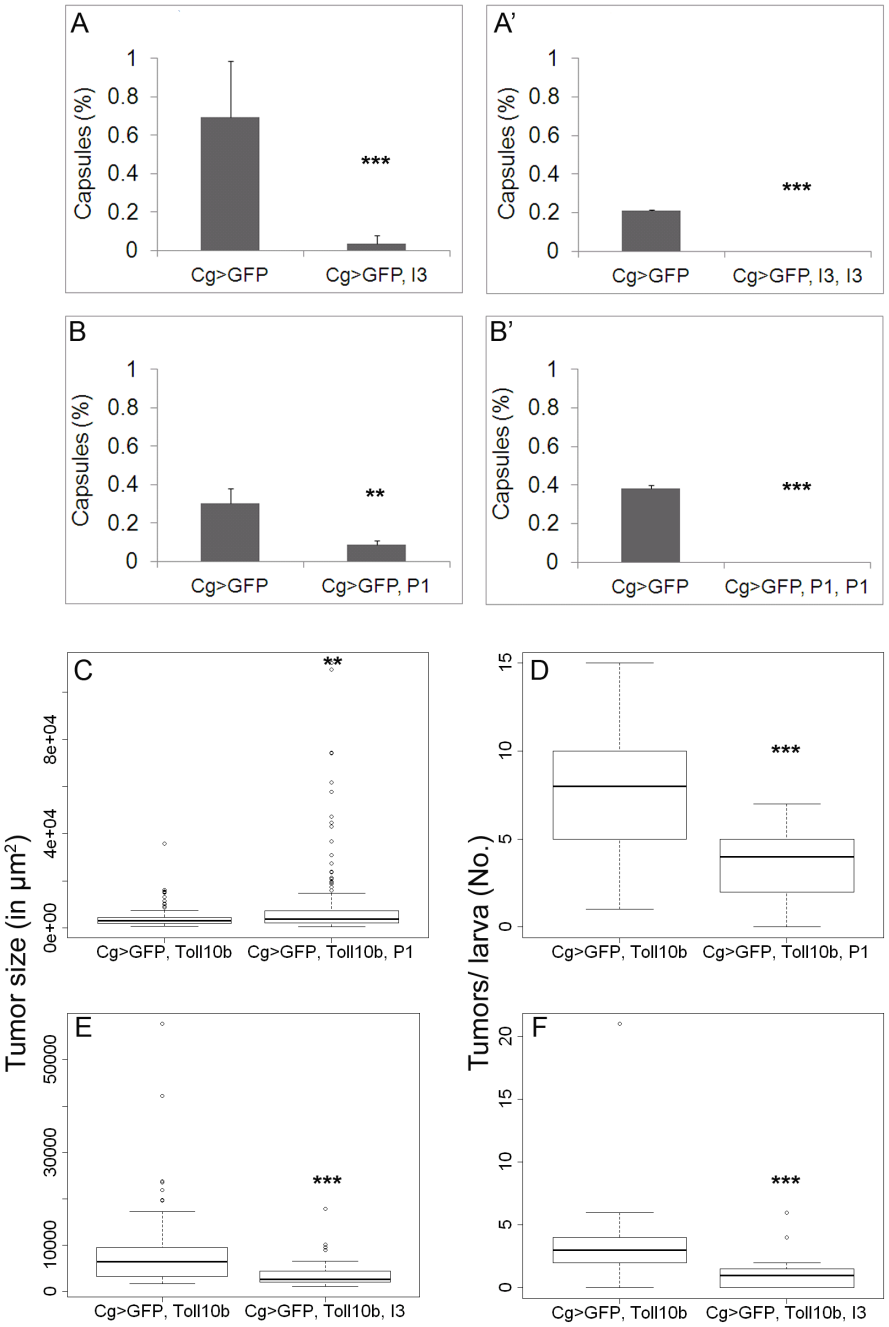
Effect of Vankyrins on wasp-induced encapsulation and tumorogenesis. **A–B′**. Decreased encapsulation when expressing Vankyrins. Comparisons are made within experiments to account for variability in percent encapsulation. **A–A′**. One copy **A** or two copies **A′** of I3 were expressed. In each case, the reduction in encapsulation was significant (X^2^ = 37.1, df = 1, p<0.001 for *Cg>GFP, I3* and X^2^ = 12.8, df = 1, p<0.001 for *Cg>GFP, I3, I3*. A. N = 54 for control and N = 46 for I3 expressing animals. A′. N = 94 for control and N = 63 for I3 expressing animals). **B–B′**. Expression of one **B**, or two copies **B′** of P1 also reduced encapsulation (X^2^ = 9.8, df = 1, p = 0.002 for *Cg>GFP, P1* and X^2^ = 115, df = 1, p<0.001 for *Cg>GFP, P1, P1*. B. N = 124 for control and N = 114 for P1 expressing animals. B′. N = 62 for control and N = 64 for P1 expressing animals). Bars indicate standard deviation. Stars indicate conditions that are different from controls (* for 0.05<p<0.01, ** for 0.01<p<0.001 and *** for p<0.001). **C–F**. Effect of Vankyrins on *Toll^10b^*-induced tumorogenesis. **C–D**. Effect of P1 on **C**. average tumor size per larva (Wilcoxon test W = 8619, p = 0.04) and **D**. average number of tumors per larva (t = −4.42, df = 21.6, p<0.001. N = 24 for control and N = 31 for P1 expressing animals). **E–F**. Effect of I3 on **E**. average tumor size (t = 53.7, df = 28, p<0.001) and **F**. average number of tumors per larva (W = 405.5, p<0.001. N = 21 for control and N = 24 for I3 expressing animals). Data are based on three independent experiments. Bars indicate standard deviation. Stars indicate conditions that are different from controls (* for 0.05<p<0.01, ** for 0.01<p<0.001 and *** for p<0.001).

We next examined if Vankyrins block immune signaling in models of acute and chronic inflammation. Exposure of fly larvae to *L. victoriae* elicits strong encapsulation [2]. Wasp infection induces limited blood cell division and differentiation and cells of the lymph gland and circulation are mobilized to encapsulate the wasp egg [6] [30]. This systemic immune reaction is resolved within hours after infection and is akin to acute inflammation in its requirement for NF-κB pathway components [2]. *Cg>GFP* larvae, with 0, 1 or 2 copies of P1 or I3 were infected with *L. victoriae* and levels of encapsulation were compared (Fig. 7). All four Vankyrin-expressing lines showed a significant decrease in their ability to encapsulate wasp embryos compared to the controls (Fig. 7A–B′). Lines expressing two copies of either P1 or I3 were completely immune-compromised and unable to encapsulate *L. victoriae* eggs (Fig. 7A′, B′).

Continuous expression of Toll^10b^ protein leads to the growth of chronic inflammatory hematopoietic tumors; this overgrowth is supported by extra rounds of mitosis triggered by Toll^10b^ expression (Fig. 7C–F, Fig. S3B). Co-expression of either P1 or I3 inhibited mitosis down to wild type levels (Fig. S3B), shrinking growth and abundance of these inflammatory hematopoietic tumors: both the size and number of microtumors per animal induced by *Cg>GFP, Toll^10b^* showed significant reduction (Fig. 7C–F, Fig. S3C–E′). Furthermore, melanization of some of the largest microtumors induced in *Cg>GFP, Toll^10b^* animals was reduced by Vankyrin expression (Fig. S3C–E′). These results suggest that Vankyrins block tumor growth by interfering with Toll^10b^-dependent pro-mitotic signals.

### Effects of Vankyrins on humoral gene expression

Two BV ankyrins (Ank-H4 and Ank-N5) from the wasp *Microplitis demolitor* reduce the ability of Dif to bind to κB consensus sequence in the *Drosomycin* promoter [17]. Biochemical studies show strong binding of Ank-H4 and Ank-N5 to homodimers of Dif and Dorsal [31]. We therefore investigated the *in vivo* effects of Vankyrins on the expression of *Drosomycin*, a direct target of Dif and Dorsal, and three *ProPO* genes, involved in melanization.

**Figure 8:**
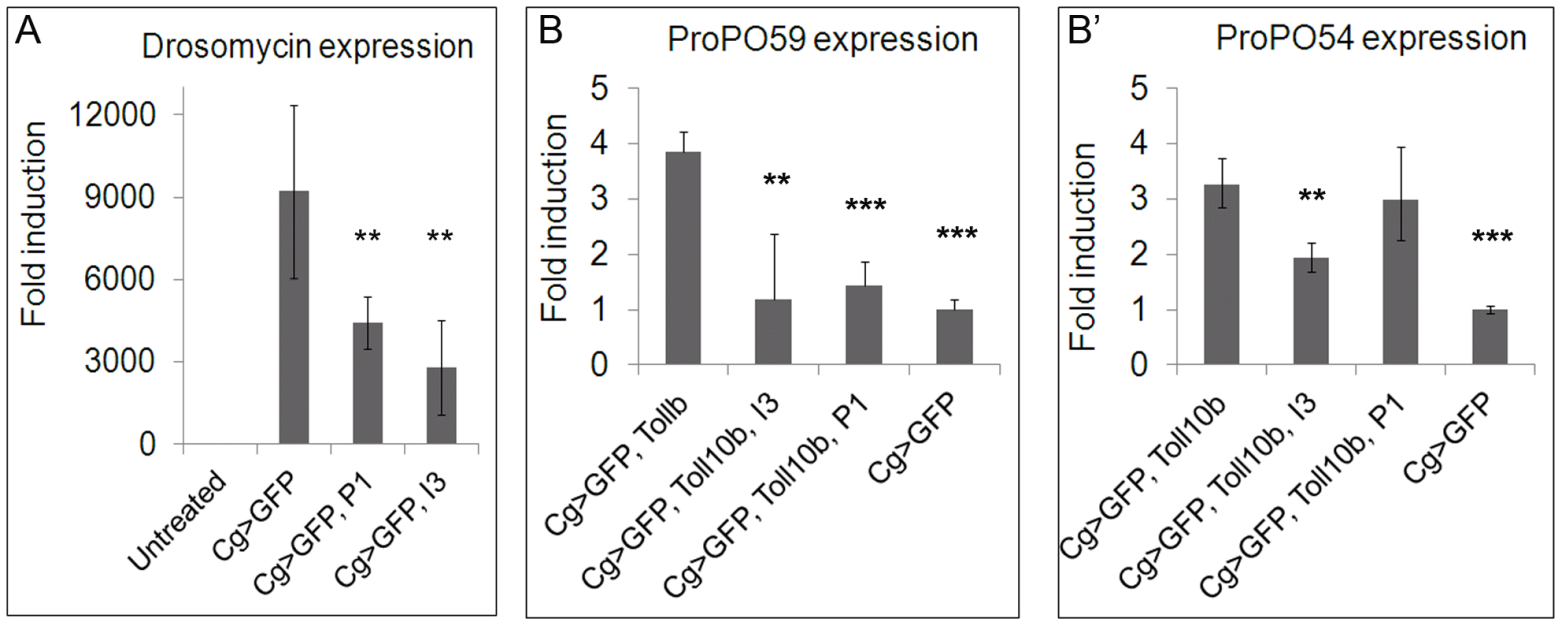
Effect of Vankyrins on immune gene expression. **A**. Both Vankyrins strongly reduce the expression of *Drosomycin* in manually-poked larvae (t = 4.1, df = 6, p = 0.006 for *Cg>GFP, P1* and t = 4, df = 5, p = 0.009 for *Cg>GFP, I3*). **B–B′**. Levels of ProPO transcripts are increased in *Cg>GFP, Toll^10b^* animals compared to controls (t = 14.2, df = 5, p<0.001 for ProPO59 and t = 11.2, df = 3, p = 0.001 for *ProPO54*). **B**. *ProPO59* expression level is reduced to control levels by expression of I3 (*Cg>GFP, Toll^10b^, I3*) (t = 3.4, df = 6, p = 0.01 compared to *Cg>GFP, Toll^10b^* and t = 0.4, df = 5, p = 0.7 compared to *Cg>GFP*). Expression of P1 (*Cg>GFP, Toll^10b^, P1*) also decreases (t = 6.9, df = 6, p<0.001 compared to *Cg>GFP, Toll^10b^* and t = 1.96, df = 5, p = 0.1 compared to controls) the expression of *ProPO59* to control levels. **B′**. The levels of *ProPO54* transcripts are only affected by I3 expression (*Cg>GFP, Toll^10b^, I3*) (t = 5.1, df = 5, p = 0.003) but not by P1 expression (*Cg>GFP, Toll^10b^, P1*) (t = 0.5, df = 5, p = 0.6). Stars indicate conditions that are different from controls (* for 0.05<p<0.01, ** for 0.01<p<0.001 and *** for p<0.001).

*Drosomycin* is highly induced in larvae poked manually with a glass needle, compared to unchallenged controls (t = 34, df = 5, p<0.001). In the presence of either Vankyrin, *Drosomycin* expression is reduced compared to challenged larvae without Vankyrins (Fig. 8A). A similar trend is observed after wasp infection, although the induction was significantly more variable (data not shown).

We also examined the effect of Toll^10b^ on the transcription of pro-phenol oxidase-encoding genes *ProPO59*, *ProPO54* and *ProPO45* [32], [33]. *ProPO59* and *ProPO54* were upregulated in *Cg>GFP, Toll^10b^* animals compared to *Cg>GFP* (Fig. 8A), while *ProPO45* was not (data not shown). Either P1 (*Cg>GFP, Toll^10b^, P1*) or I3 (*Cg>GFP, Toll^10b^, I3*) reduced ProPO59 expression compared to the control *Cg>GFP* levels (Fig. 8B). I3 expression (*Cg>GFP, Toll^10b^, I3*) reduced the expression of *ProPO54* relative to *Cg>GFP, Toll^10b^* larvae, P1 expression (*Cg>GFP, Toll^10b^, P1*) did not have this effect (Fig. 8B′).

### P1 and I3 enhance the haploinsufficiency of *dl*

**Figure 9:**
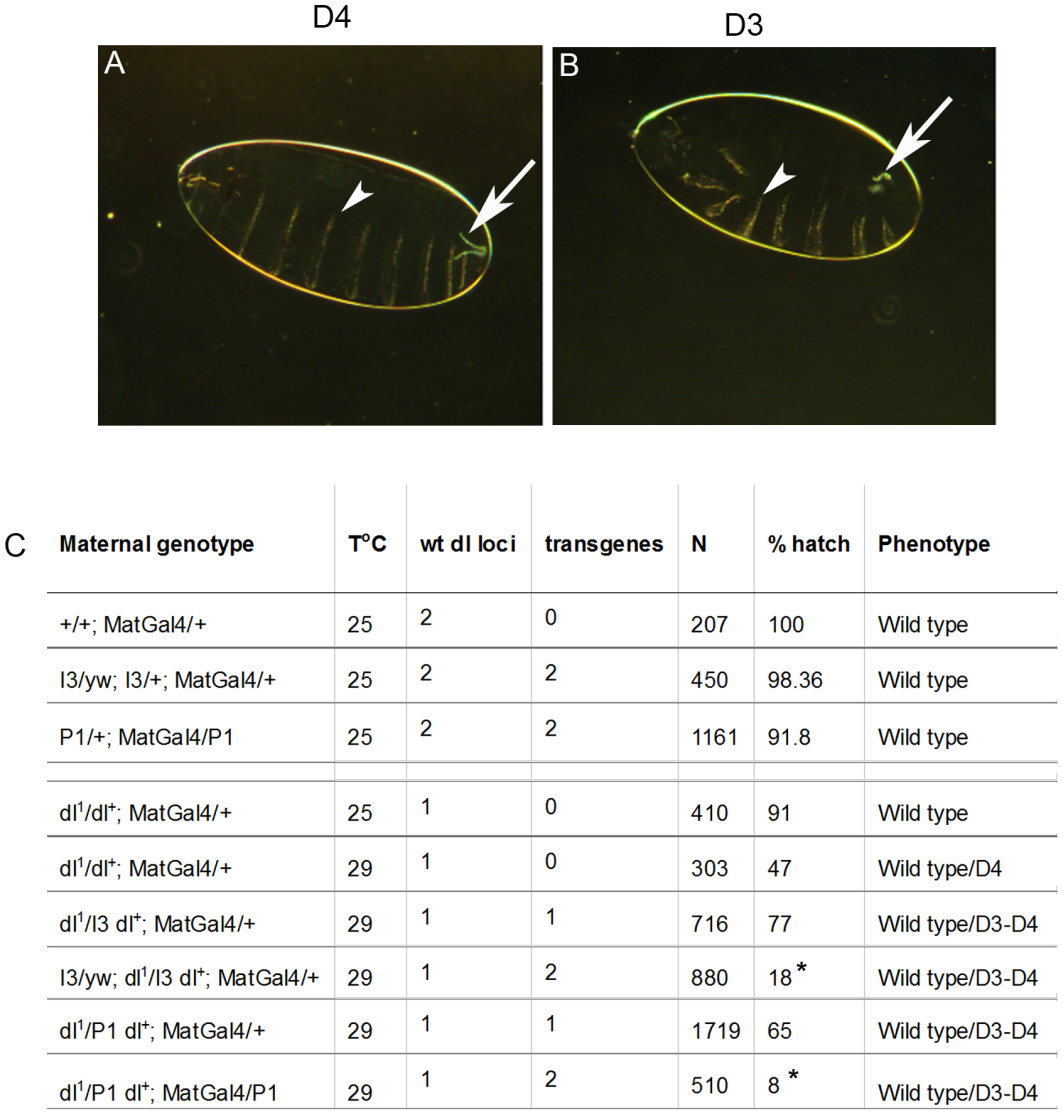
Vankyrins enhance maternal *dorsal* haplo-insufficiency in early embryonic development. **A–B**. Phenotypes of dorsalized embryos imaged in dark field. **A**. Embryo with weak dorsalization defects (D4). **B**. Embryo with somewhat stronger dorsalization showing reduction of ventral denticle belts (D3). Arrows point to the filzkorper. Arrowheads point to the ventral setae. Neither D4 not D3 embryos hatch. **C**. Percentage of hatching embryos for each maternal genotype and associated phenotypes. Maternal genetic background for the *dl* locus was either wild type or heterozygous *dl^1^/+*. A significant decrease in the percentage of hatching was observed for animals expressing two copies of either P1 (t = 5.5, df = 2.37, p = 0.02) or I3 (t = 4.02, df = 2.6, p = 0.036) compared to embryos from *dl^1^/+* control females at 29°C. The percentages were not significantly affected when only one copy was expressed (t = −1.9, df = 4.69, p = 0.12 for P1 and t = −2.9, df = 2.3, p = 0.087 for I3). N represents the total number of fertilized embryos analyzed; unfertilized embryos were not included. Stars indicate conditions that are different from controls (* for 0.05<p<0.01, ** for 0.01<p<0.001 and *** for p<0.001).

The Toll pathway specifies dorsal-ventral fates during early embryogenesis. Embryos lacking maternal *dl* become dorsalized. To examine the effects of vankyrins on d/v patterning, we took advantage of the temperature-dependent haploinsufficiency of *dl* [34]. At 29°C, only 47% of the embryos derived from heterozygous *dl^1^/+* females hatch, while the remaining, unable to develop normally, show slight dorsalization (Fig. 9A). We introduced 1 or 2 copies of each Vankyrin using the maternal driver Mat-Gal4. With only one copy of either P1 or I3 in *dl^1^/+* females, the percent hatch did not differ significantly from the control (Fig. 9A). But with two copies of P1, only 8% of the embryos hatched, and, with two copies of I3, 18% of the embryos hatched. In both cases, the degree of dorsalization of unhatched embryos is more severe such that the ventral denticle belts, markers of ventral fate, are visibly reduced (Fig. 9A, B). Although, Mat-Gal4>P1 or Mat-Gal4>I3 in wild type background do not have strong effects; P1 expression has a mild and general effect on embryonic development. These results suggest that immune-suppressive Vankyrins can also block Toll signaling in the embryo and that multiple copies of *vankyrin* genes in PDV genomes ensure physiological and specific inhibition.

## Discussion

Despite common features, mutualistic polydnaviruses of Braconid and Ichneumonid wasps derive from different ancestral viruses [19], [20]. Yet, genomes from both families encode several copies of *vankyrin* genes. While high sequence similarity and multiple gene copies in the PDV genome may suggest similar localization or redundant biological effects, we observed surprising differences in localization of I3 and P1, and qualitative and quantitative differences of their effects on NF-κB signaling in hematopoiesis, immunity, and development.

### Parasite infection is sensed by the niche and it reprograms hematopoietic development to support parasite egg encapsulation

The role of NF-κB proteins in larval hematopoiesis has been an open question for over a decade. Here we have uncovered a novel role for NF-κB signaling in the niche, where it controls the proper proportion of mature hematopoietic lineages. This discovery is validated by inhibitory effects of I^2^-vank-3. Dif and Dorsal are expressed in the niche. Excessive Dorsal or Dif in the niche is sufficient to trigger constitutive differentiation and release of lamellocytes. While single RNAi knockdowns have weak effects, genetic removal of both *Dif* and *dl* encourages supernumerary crystal cells and also reduces wasp-induced lamellocytes (our unpublished results). Thus, it seems that moderate levels of NF-κB activity (not essential either for Antp expression or for the specification of a particular lineage) are required for gene expression in the niche to guarantee the correct ratio of specific lineages. Elevated or diminished NF-κB activity perturb this balance: high levels parallel conditions of wasp infection (more lamellocytes; fewer crystal cells; this study [4]) whereas low levels have the opposite effect (this study). These data synthesize our view of how wasp infection shifts the balance of NF-κB functions, favoring lineage development for egg encapsulation. The identities of NF-κB target genes and their effects on lineage commitment remain to be discovered.

This interpretation that basal versus activated hematopoieses support distinct lineage programs suggests that cells of the niche, very likely, sense the systemic environment of the larval hemocoel, and respond to the host's immune status by switching states. It is therefore reasonable to conclude that the mechanism governing this switch includes NF-κB signaling itself, whose activation state depends directly on the infection status (this study; [3], [7]). How is infection sensed by the niche? Recent work suggests that the Toll ligand, Spätzle (*spz*) and Spätzle-processing enzyme (SPE cleaves and activates inactive Spz) are involved. (1) Spz protein and *SPE* transcripts are expressed at high levels in circulating blood cells and uniformly in cells of the lymph gland lobes [2], [35], [36]. (2) Spz and SPE expression is activated by wasp infection in both compartments [2]. (3) Mis-expression of either transgene in blood cells, or even just in the fat body, induces lamellocyte differentiation and systemic inflammation [2]. (4) The niche also senses the animal's nutritional status [37]. Thus, it appears that the niche is functionally flexible and responds to hemolymph factors by reprogramming hematopoiesis.

The observation that niche-specific expression of I3 alone reduces niche cell count, reduces Antp>GFP expression, and modulates both basal and activated hematopoiesis suggests that NF-κB signaling has complex and specific transcriptional effects that directly and indirectly control multiple parameters of niche function in response to organismal physiology. A lack of inhibition by P1 suggests that I3 may be a better inhibitor of Dif, which is expressed more strongly in the niche relative to other parts of the lobe. It is not surprising, then, that immune-suppressive viruses aiding parasite survival are poised to paralyze NF-κB signaling in hematopoiesis regardless of its activation status.

### Selective inhibition of NF-κB signaling by viral ankyrins

It is of interest that P1 and I3 share 83% amino acid identity and yet have strikingly different biological effects: (1) In basal and activated lymph gland hematopoiesis, I3 promotes crystal cell development, P1 does not; (2) their expression in circulating cells (via Cg>Gal driver) does not alter blood cell counts, but Antp>I3 encourages crystal cell development in sessile and circulating cell compartments; (3) both I3 and P1 reduce wasp egg encapsulation; (4) both Vankyrins block Cg>Toll^10b^-induced mitosis and resulting tumorogenesis, antifungal peptide gene expression and *proPO* gene expression (although I3's inhibitory effect on *ProPO54* expression is stronger than that of P1's); and (5) in embryogenesis, P1's effects on dorsalization are stronger than those of I3's. We explain these differences on NF-κB signaling by postulating pre-existing differences in concentration and localization of a particular Vankyrin and NF-κB protein(s) (fly embryos do not express Dif) and their complexes in different cell types.

Differences in sub-cellular localization between P1 and I3 offer additional clues. Both P1 and I3 are cytoplasmic and punctate in blood cells from uninfected larvae, but on infection, both proteins assume a nuclear bias, co-localizing with GFP-Dorsal. While I3 and GFP-Dorsal appear vesicular and perinuclear, P1 and GFP-Dorsal co-localize within the nucleus. Recent experiments in the fly embryo [38] show that endocytosis is central to Toll signaling. It is, thus, possible that I3 interaction in blood cells with Dorsal blocks its endocytosis and/or nuclear uptake. P1 additionally may inhibit transcriptional activation of GFP-Dorsal. We noted, in these staining experiments, significant changes not only in their sub-cellular localization in resting versus immune-active cells, but also in their amounts. We interpret that Vankyrins themselves are subject to translational and/or post-translational regulation, a conclusion that is supported by measurements in transgenic cell culture studies [28]. Specificities in translational and/or post-translational regulation in different cell types may result in different biological outcomes.

Finally, like Bracovirus ankyrins H4 and N5, that bind to Dorsal and Dif, with different affinities [31], Ichnoviral Vankyrins appear to have differential affinities for NF-κB proteins. Our data suggest that I3 may have a preference for Dif, whereas P1 may bind more strongly to Dorsal in the absence of Dif.

### Conclusions

Parasitoid wasps make up thousands of species. Using *Drosophila* and its natural parasitic wasps we have shown that hematopoiesis and the cellular egg encapsulation response are tightly-linked via NF-κB signaling. NF-κB signaling is active in the niche in the absence of wasp infection, but wasp infection activates NF-κB signaling further and reprograms hematopoiesis for wasp egg encapsulation. Our data suggest that even highly identical Ichnoviral ankyrins, I^2^-vank-3 and P-vank-1, perturb cellular and humoral immunity with remarkable specificity to contribute to the success of their wasp, *C. sonorensis*. Our results predict that parasitoid infections activate both immune arms of their natural insect hosts, NF-κB-IκB interactions underlie this activation, and successful immune-suppression targets both immune arms. The *Drosophila* model system can be used to explore molecular functions of additional immune-suppressive molecules critical to species survival and evolution of natural communities.

Targeting immune pathways of enemies by immune-suppressive molecules is a general strategy for success among Hymenopteran insects [36]. Components in the bee venom protect bees against arthropod and vertebrate predators. Bee venom is an ancient therapy for chronic inflammation and pain relief [39]. The context-independent inhibitory effects of Vankyrins on NF-κB signaling reported here provide one clear mechanism by which anti-inflammatory effects of Hymenopteran products may be realized. In addition, inhibition of NF-κB signaling continues to be a significant area of research for strategic development of drug targets for human diseases [40], [41]. Detailed structural studies coupled with rational design of IκB-family ankyrin repeats have potential for the treatment of inflammation-based human diseases from arthritis to cancer. They can also provide the means to weaken the immune system of insect pests to improve agriculture and human health [42].

## Materials and Methods

### Strains and crosses

All *D. melanogaster* stocks were raised on standard medium at 25°C. Standard crosses were performed to obtain the desired genetic backgrounds. *y w; Ubc9/CyO y^+^* [14] lines were used for anti-Dif and anti-Dorsal staining. Gal4 lines: *Cg-Gal4, UAS-GFP* (expressed in fat body, lymph gland and hemocytes), *Hml-Gal4, UAS-GFP* (expressed in hemocytes and lymph gland), *y w; UAS-mcd8GFP; Antp-Gal4/TM6 Tb* (expressed in the lymph gland niche; abbreviated as *Antp>GFP*), *Srp-Gal4, GFP-dl* (expressed in hemocytes). UAS lines: *UAS-dl* (S. Tanda, Ohio State Univ.), *UAS-Toll^10b^* (constitutively active Toll receptor), *UAS-dl^RNAi^* (TRiP), *UAS-Dif^RNAi^* (VDRC, transformant 30579), *UAS-GFP* (Bloomington Stock Center) and the *UAS-vankyrin* and *UAS-Dif* lines as below. Reporter strain for Dorsal/Dif activity, *D4-lacZ* (A. Courey) contains four tandemly repeated Dorsal/Dif binding sites [27].

To induce flp-out clones [43], developmentally-synchronized 4-day old larvae with the hybrid flip-out and Gal4 activation system [*hsp70-flp*; *Actin*>*CD2*>*Gal4*] and *UAS*-*GFP* transgenes or those with an additional *UAS-GFP-dl* transgene, were heat-shocked at 37°C in a water bath for 15 min.

To examine their effects on embryonic development, Vankyrins were expressed maternally using the *dl^1^/CyO; Mat-Gal4* strain (A. Courey, UCLA). Percentage of eggs (n = 300 or more) hatched from females with 0, 1, or 2 copies of either *P1* or *I3* transgenes was recorded.

Mutant strains: *b dl^8^/CyO b; y w; Df(2L)TW119/CyO y^+^* and *y w; Df(2L)J4/CyO y^+^* [14].

### Production of transgenic lines

*Vankyrin* cDNAs from *C. sonorensis*, P-vank-1 (P1, Accession: AAX56953.1, 171 amino acids) and I^2^-vank-3 (I3, Accession AAX56959.1, 171 amino acids) (kindly provided by Dr B. Webb, University of Kentucky [16]), were amplified by PCR using forward primers containing a FLAG tag and an EcoR1 restriction site and a common reverse primer containing a Xba1 restriction site (Text S1).

Both cDNAs were cloned into the P-element containing vector pUAST. This vector contains five GAL4 binding sites, allowing GAL4 inducible expression of vankyrins [44]. Constructs were injected into *y w* embryos (Rainbow Transgenic Flies, Camarillo, California, USA). A strain bearing the *UAS-Dif* transgene was constructed by inserting the full-length *Dif* cDNA [45] into the pUAST vector and injections were done in-house.

### Wasp infection

*L. victoriae* or *L. boulardi* adults were exposed to developmentally-synchronized larvae. Two days after infection, fly larvae were dissected to score for infection and encapsulation.

### Effects of Vankyrins on tumors and encapsulation

Fly larvae from a 48-hour egg lay expressing either *vankyrin* cDNAs were infected by *Leptopilina victoriae* for 24 hours. After 48 hours, larvae were dissected and the number of live and encapsulated wasp larvae was recorded. To test dose response, larvae with either one copy (*Cg>P1* or *Cg>I3*) or two (*Cg>P1, P1* or *Cg>I3, I3*) copies of *vankyrin* transgenes were used. *Cg-Gal4, UAS-GFP* flies were used as controls.

To evaluate immune suppressive effects of Vankyrins on the Toll pathway, *Cg>Toll^10b^* (control) and *Cg>Toll^10b^, P1* or *Cg>Toll^10b^, I3* larvae were examined for tumor penetrance and expressivity. Third instar larvae from a 6 hour egg-lay were dissected and blood cells, aggregates and tumors from their hemolymph were stained for DNA (Hoechst 33258 – Molecular Probes) and F-actin (rhodamine phalloidin – Invitrogen). Images were acquired using a Zeiss Axioscope 2 Plus fluorescence microscope. The size and number of tumors were recorded using AxioVision LE 4.5 software.

### Immuno-histochemistry

Developmentally synchronized larvae were collected, washed and dissected for either hemolymph or lymph gland according to methods described previously [23]. Antibodies and dilutions used are as follows: β-galactosidase (chicken anti-β-Gal, 1∶200; Immunology Consultants Laboratory, Inc.), rabbit anti-phosphohistone H3 (1∶200 Upstate), mouse anti-prophenoloxidase (anti-ProPO, 1∶10; Dr. T. Trenczek, University of Giessen) or rabbit anti-ProPO2 [46], mouse anti-Dorsal (anti-dorsal 7A4, 1∶4; DHSB, Iowa [47]), rabbit anti-Dif (1∶500; Dr D. Ferrandon – IBMC, Strasbourg), mouse anti-Antp (8C11, 1∶20; DSHB, Iowa), and mouse anti-L1/Atilla (1∶10, I. Ando [48]), mouse anti-Integrin β PS (CF6G11, 1∶10; DSHB, Iowa), and mouse anti-FLAG (1∶1000; Sigma). Secondary antibodies were Cy5, Cy3 or Alexa647 anti-mouse (1∶200; Jackson Immunological and 1∶1000, Invitrogen, respectively), Cy3 anti-chicken (1∶500; Jackson Immunological) and FITC or Cy3 conjugated anti-rabbit (1∶200; Jackson Immunological). All samples were counterstained with Hoechst 33258 (Molecular Probes). Rhodamine phalloidin (Invitrogen) was used where indicated.

### Imaging, data collection, and analysis

#### Confocal imaging

Images were acquired using a Zeiss Laser Scanning Confocal microscope; a scale bar was added with Zeiss LSM5 software. A series of consecutive confocal optical sections (Z-stacks) were recorded at 8 bit. Figures were assembled in Photoshop CS6 (Adobe Systems Inc., San Jose, CA). All samples for the experiment were scanned with the same microscope and software settings. Image acquisition settings were adjusted to avoid under- or over-exposure by limiting white and black clipping to less than 1% of pixels. In these settings, a majority of pixels in the cells of interest had signal intensity between 0–255 (8 bit). Images where a high number of pixels were not detected (underexposed) or were saturated (overexposed) were not used for quantification. A negative control (i.e., sample treated with secondary antibody but without primary antibody to detect non-specific signal) was used to establish an amplifier gain/offset cut-off value.

#### Intensity measurements in the niche

For analysis in Adobe Photoshop CS6, Z-stacks of images were exported from LSM 510 software (one color at a time) to tif format without compression and in 8 bit, as series. The Elliptical Marquee Tool was used to select an ellipse of constant area. Elliptical regions were randomly selected over at least 15 cells (or all cells if the total number of niche cells is below 15) through all optical sections of a Z-stack. The Histogram Tool quantifies and averages 0–255 range of signal intensity from the selected area and this tool was used to collect data from over 1,000 pixels per niche. Intensity values were compared for matched experimental groups. Average pixel intensity and standard deviation for each experimental group is presented in Fig. 3G and Fig. 4G.

#### Cell counts

(a) Cells in the niche were defined by Hoechst-positivity for the nucleus and cell-associated Antp>mCD8-GFP fluorescence (Fig. 3F and 4H). Optical Z-stacks were serially analyzed to count all cells. (b) For crystal cells and lamellocytes (Supplement 3F and 3L), cells positive for the anti-pro-PO or anti-integrin-beta antibodies, respectively, and characteristic morphologies, were counted. Optical Z-stacks were analyzed. (c) For the number of sessile/circulating crystal cells in three posterior-most segments, larvae were heated in PBS (70°C for 15 minutes) and were mounted on slides. Melanized cells, visible through the transparent cuticle, were counted using 100× magnification of a Leica stereomicroscope.

#### Encapsulation

Wasp-exposed or control larvae were dissected. For each, the number of wasp capsules and the number of wasp larvae were recorded under a stereomicroscope. Uninfected animals were excluded. Percent encapsulation was scored and plotted with the standard deviation (Fig. 7A–B′).

#### Blood cell counts and tumors

For cell counts, third instar animals were bled and cell concentration was determined using a hemocytometer. For tumor phenotype expressivity and penetrance, larvae were dissected on slides; the entire hemolymph preparation was fixed, stained with Hoechst and imaged for quantification. Expressivity of the tumor phenotype was measured by scoring the area of each tumor (Fig. 7C, E) using AxioVision LE 4.5 Outline Tool. Using the same images, the phenotype penetrance, *i.e.*, number of tumors per larva (Fig. 7D, F) was scored.

### Preparation of epidermal cuticles

The cuticle patterns of embryos from wild type females or haplo-insufficient for *dl* and expressing 0, 1 or 2 copies of either *vankyrin* gene were visualized after dechorionation, clearing and mounting in Hoyer's mountant. Images were acquired using dark field optics using a Zeiss Axioscope 2 Plus fluorescence microscope.

### RNA extraction and quantitative PCR

RNA was extracted from 7 to 10 pooled third instar larvae using Trizol (Invitrogen). RNA concentration and quality was checked before treating the samples with DNase (Turbo DNA-Free, Ambion). The first cDNA strand was then synthesized using ProtoScript M-MuLV First strand cDNA synthesis kit (NEB). The volume was then completed to 50 µl. RNA was stored at −80°C while cDNA was aliquoted and stored at −20°C.

For quantitative PCR, iQ SYBR Green supermix kit (Biorad) was used as per the manufacturer's recommendations except the reactions were done in 20 µl. Primers used and PCR conditions are described in Text S1. For the qPCR, three technical and 3–4 biological repeats were performed. Transcripts levels were normalized using the ribosomal *rp49* gene. Melting curves were analyzed and quantification was made by using the ΔΔCT method.

### Statistical analysis

All analysis except for qPCR were performed using the R software [49]. All data were tested for normality. The non-normal data were transformed when possible or a non-parametric test was applied. qPCR data were analyzed by Student t-test using the trial version of GenEx software (http://www.biomcc.com/genex-software.html).

## Supporting Information

Figure S1 Manipulation of Dif/Dorsal levels in the niche affects hematopoietic development. **A–F**. Crystal cells (**A–E**, magenta) were visualized with ProPO2 antibody in **A**. wild type (*wt*), **B**. *Antp>GFP, Dif^RNAi^*, **C**. Antp>GFP, *dl^RNAi^*, **D**. *Antp>GFP, Dif*, **E**. *Antp>GFP, dl* background. **F**. Crystal cell counts for each genotype. The average number of crystal cells in anterior lobes remain unchanged in *Antp>Dif^RNAi^* (t = −1.6, df = 7.7, p = 0.15), *Antp>dl^RNAi^* (t = −1.7, df = 6.6, p = 0.14 for), Antp>dl (W = 45.5, p = 0.8) and is slightly increased in Antp>Dif (t = −2.8, df = 17.67, p = 0.011), compared to controls. **G–L**. Lamellocytes, characterized by large nuclei, and with high integrin β PS staining (red, **G–K**) are rare in **G**. wild type (wt), **H**. *Antp>GFP, Dif^RNAi^*, and **I**. Antp>GFP, *dl^RNAi^*, but are abundant in **J**. *Antp>GFP, Dif*, and **K**. *Antp>GFP, dl* backgrounds. **L**. Average number of lamellocytes per anterior lobe is significantly higher in *Antp>Dif* (Wilcoxon test W = 5.5, p<0.001) and *Antp>dl* (W = 5.5, p<0.001) animals compared to controls. Cell counts represent an average per lobe for N = 5 animals per genotype. Stars indicate statistical significance relative to controls (* for 0.05<p<0.01, ** for 0.01<p<0.001 and *** for p<0.001). *Antp>GFP* expression visible in panels **A–E** (or G–K) of this figure is also presented at higher magnification in Fig. 3.
(TIF)

Figure S2 Flp-out clones with GFP-Dorsal support non cell-autonomous effects on hematopoietic lineage development. **A–B**. Lymph glands with flp-out clones which express GFP (**A**, green), or GFP and GFP-Dorsal (**B**, green) stained for Pro-PO2 (crystal cells, magenta, **A–B**). Insets (**A–B**) show magnified crystal cells neighboring the clones. **C**. A reduction in crystal cells per pair of anterior lobes was found in lymph glands expressing GFP-Dorsal (t = −2.5, df = 30.1, p = 0.02. N = 18 for controls; N = 22 for GFP-Dorsal-expressing animals). **D–E**. Lymph glands with flp-out clones which express GFP (**D**, green), or GFP and GFP-Dorsal (**E**, green) stained with anti-Integrin-β PS to mark lamellocytes (**D–E**, red). **F**. Supernumerary lamellocytes are observed in glands with GFP-Dorsal clones. **E**, but not in glands with control clones, **D**. (Wilcoxon test W = 55.5, p<0.001. N = 8 for control; N = 7 for GFP-Dorsal-expressing animals). Stars indicate conditions that are different from controls (* for 0.05<p<0.01, ** for 0.01<p<0.001 and *** for p<0.001).
(TIF)

Figure S3 Effects of Vankyrins on circulating blood cell population, and on mitosis and tumors due to hyperactive Toll activity. **A**. *Cg>GFP* driven Vankyrin (2 copies each of P1 or I3) expression does not significantly affect circulating hemocyte concentration (CHC). In all cases, CHC is within the control range [7]. Comparison between control *Cg>GFP* and *Cg>GFP, P1* larvae: (11,585±3,131 cells versus 12,966±2,374 cells; t = −1.4, df = 27.96, p = 0.17. N = 16) and *Cg>GFP* versus *Cg>GFP, I3* larvae: (11,585±3,131 cells versus 12,539±2,612 cells; t = −0.9, df = 29.07, p = 0.36. N = 16). All cells in all three genotypes are GFP-positive. **B**. Expression of Cg>Toll^10b^ increases the proportion of phospho-histone H3 (PH3)-positive cells (Wilcoxon test W = 10.5, p<0.01. N = 8 for *Cg>GFP*; N = 14 for *Cg>GFP, Toll^10b^*), while Vankyrin expression reverses this effect (Wilcoxon test W = 4.5, p<0.01 for *Cg>Toll^10b^, GFP* compared to *Cg>Toll^10b^, GFP, P1*; Wilcoxon test W = 7.5, p<0.01 for *Cg>Toll^10b^, GFP* compared to *Cg>Toll^10b^, GFP, I3*. N = 14 for *Cg>Toll^10b^, GFP*; N = 11 for *Cg>Toll^10b^, GFP, P1* and N = 27 for *Cg>Toll^10b^, GFP, I3*). Vankyrin expression reverses mitosis to control levels (Wilcoxon test W = 42.5, p = 0.86 for *Cg>GFP* compared to *Cg>Toll^10b^, GFP, P1* and Wilcoxon test W = 94.5, p = 0.61 *Cg>GFP* compared to *Cg>Toll^10b^, GFP, I3*. N = 8 for *Cg>GFP*; N = 11 for *Cg>Toll^10b^, GFP, P1* and N = 27 for *Cg>Toll^10b^, GFP, I3*). **C–E′**. Melanized tumors from **C–C′** *Cg>GFP, Toll^10b^*, **D–D′** *Cg>GFP, Toll^10b^ P1*, or **E–E′** *Cg>GFP, Toll^10b^ I3* larvae.
(TIF)

Text S1 Nucleotide sequences of primers used for polymerase chain reaction amplification conditions (number of amplification cycles and melting temperatures) used for different genes considered in this study.
(DOC)
