# Supplementary figures and images for: Polydnaviral Ankyrin Proteins Aid Parasitic Wasp Survival by Coordinate and Selective Inhibition of Hematopoietic and Immune NF-kappa B Signaling in Insect Hosts
Source: PLoS Pathog. 2013 Aug 29;9(8):e1003580. doi: 10.1371/journal.ppat.1003580 (PMC3757122; doi:10.1371/journal.ppat.1003580)

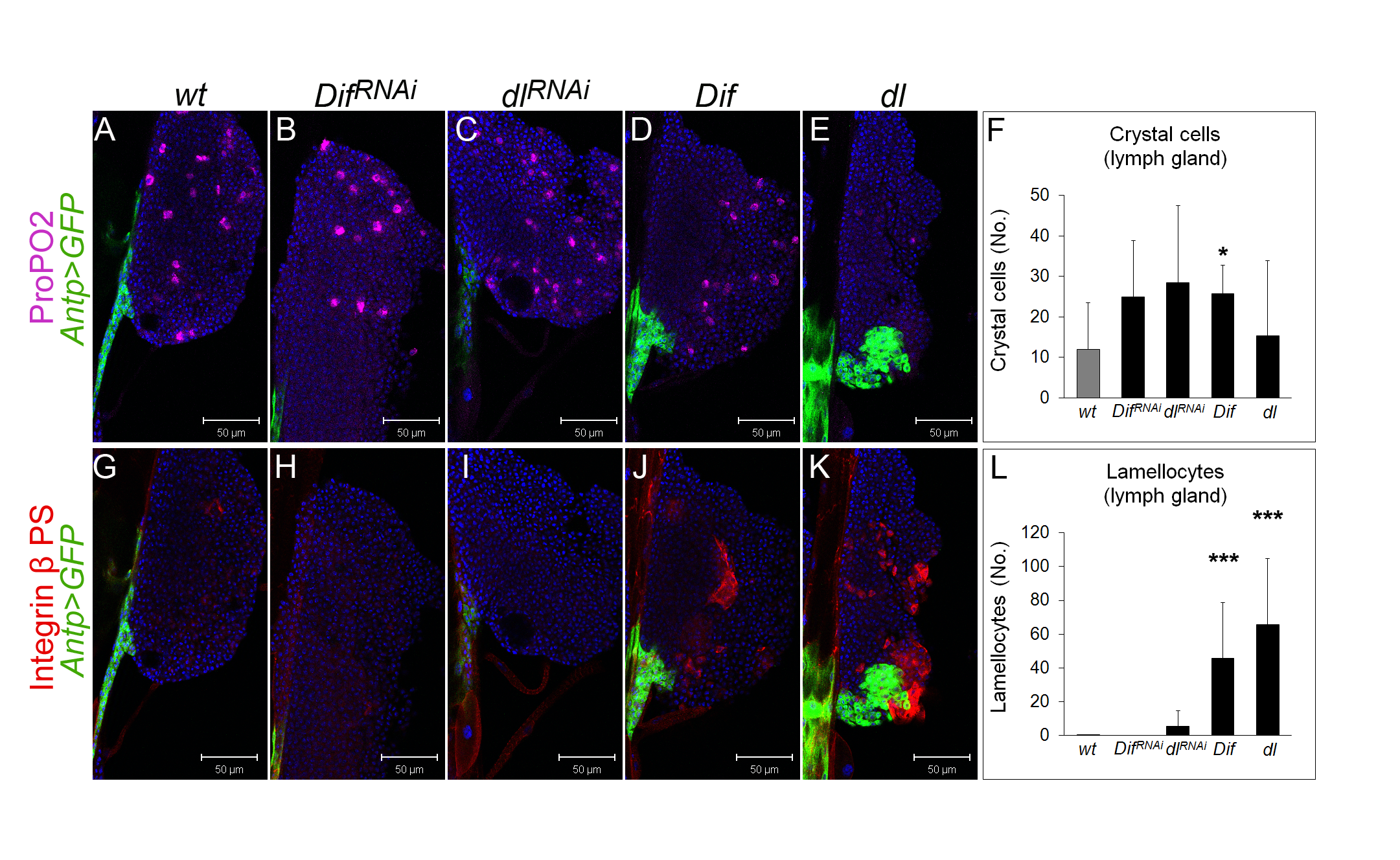

Supplement: Figure S1 — Manipulation of Dif/Dorsal levels in the niche affects hematopoietic development. A–F. Crystal cells (A–E, magenta) were visualized with ProPO2 antibody in A. wild type (wt), B. Antp>GFP, DifRNAi, C. Antp>GFP, dlRNAi, D. Antp>GFP, Dif, E. Antp>GFP, dl background. F. Crystal cell counts for each genotype. The average number of crystal cells in anterior lobes remain unchanged in Antp>DifRNAi (t = −1.6, df = 7.7, p = 0.15), Antp>dlRNAi (t = −1.7, df = 6.6, p = 0.14 for), Antp>dl (W = 45.5, p = 0.8) and is slightly increased in Antp>Dif (t = −2.8, df = 17.67, p = 0.011), compared to controls. G–L. Lamellocytes, characterized by large nuclei, and with high integrin β PS staining (red, G–K) are rare in G. wild type (wt), H. Antp>GFP, DifRNAi, and I. Antp>GFP, dlRNAi, but are abundant in J. Antp>GFP, Dif, and K. Antp>GFP, dl backgrounds. L. Average number of lamellocytes per anterior lobe is significantly higher in Antp>Dif (Wilcoxon test W = 5.5, p<0.001) and Antp>dl (W = 5.5, p<0.001) animals compared to controls. Cell counts represent an average per lobe for N = 5 animals per genotype. Stars indicate statistical significance relative to controls (* for 0.05<p<0.01, ** for 0.01<p<0.001 and *** for p<0.001). Antp>GFP expression visible in panels A–E (or G–K) of this figure is also presented at higher magnification in Fig. 3. (TIF) [file ppat.1003580.s001.tif]

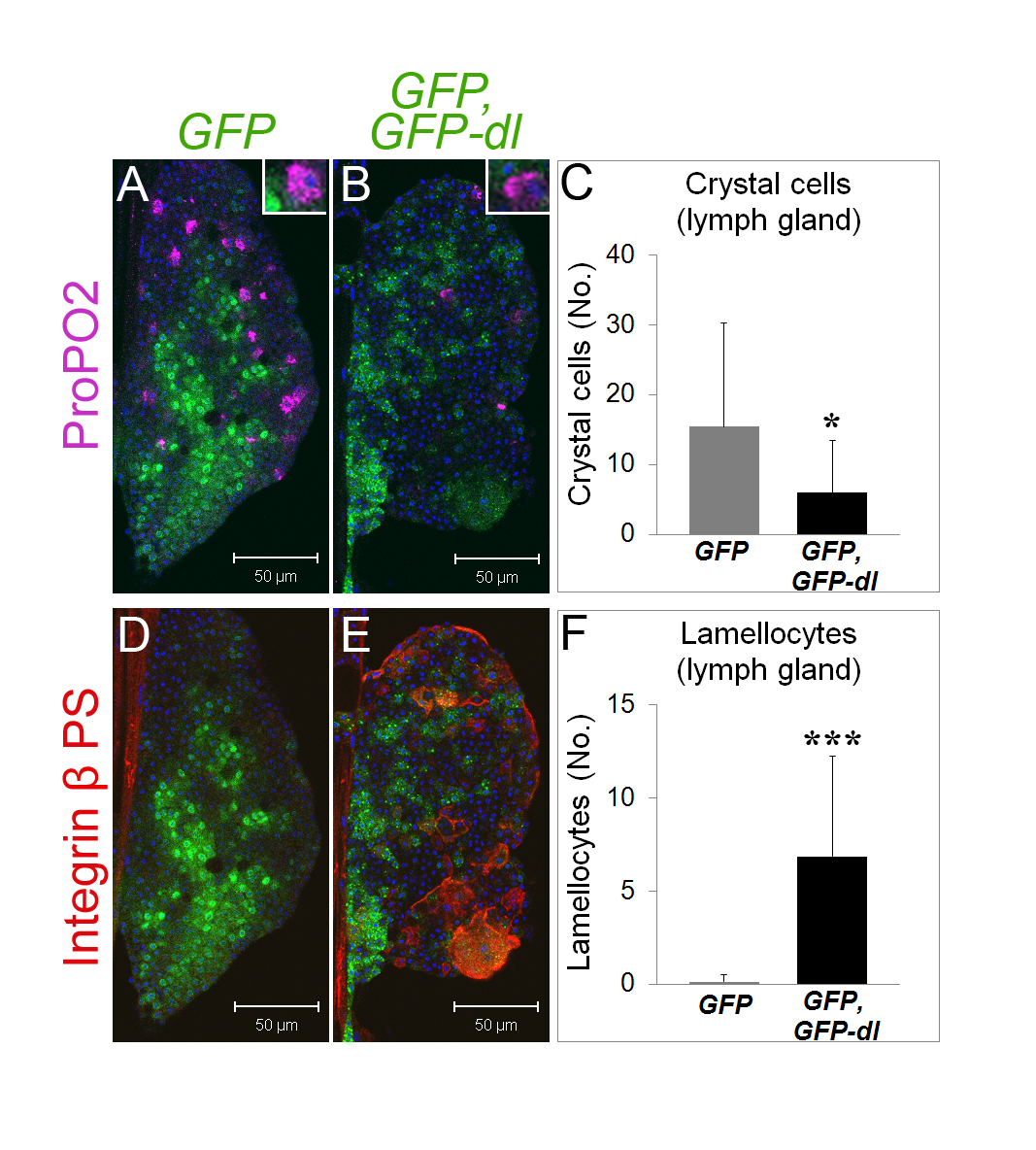

Supplement: Figure S2 — Flp-out clones with GFP-Dorsal support non cell-autonomous effects on hematopoietic lineage development. A–B. Lymph glands with flp-out clones which express GFP (A, green), or GFP and GFP-Dorsal (B, green) stained for Pro-PO2 (crystal cells, magenta, A–B). Insets (A–B) show magnified crystal cells neighboring the clones. C. A reduction in crystal cells per pair of anterior lobes was found in lymph glands expressing GFP-Dorsal (t = −2.5, df = 30.1, p = 0.02. N = 18 for controls; N = 22 for GFP-Dorsal-expressing animals). D–E. Lymph glands with flp-out clones which express GFP (D, green), or GFP and GFP-Dorsal (E, green) stained with anti-Integrin-β PS to mark lamellocytes (D–E, red). F. Supernumerary lamellocytes are observed in glands with GFP-Dorsal clones. E, but not in glands with control clones, D. (Wilcoxon test W = 55.5, p<0.001. N = 8 for control; N = 7 for GFP-Dorsal-expressing animals). Stars indicate conditions that are different from controls (* for 0.05<p<0.01, ** for 0.01<p<0.001 and *** for p<0.001). (TIF) [file ppat.1003580.s002.tif]

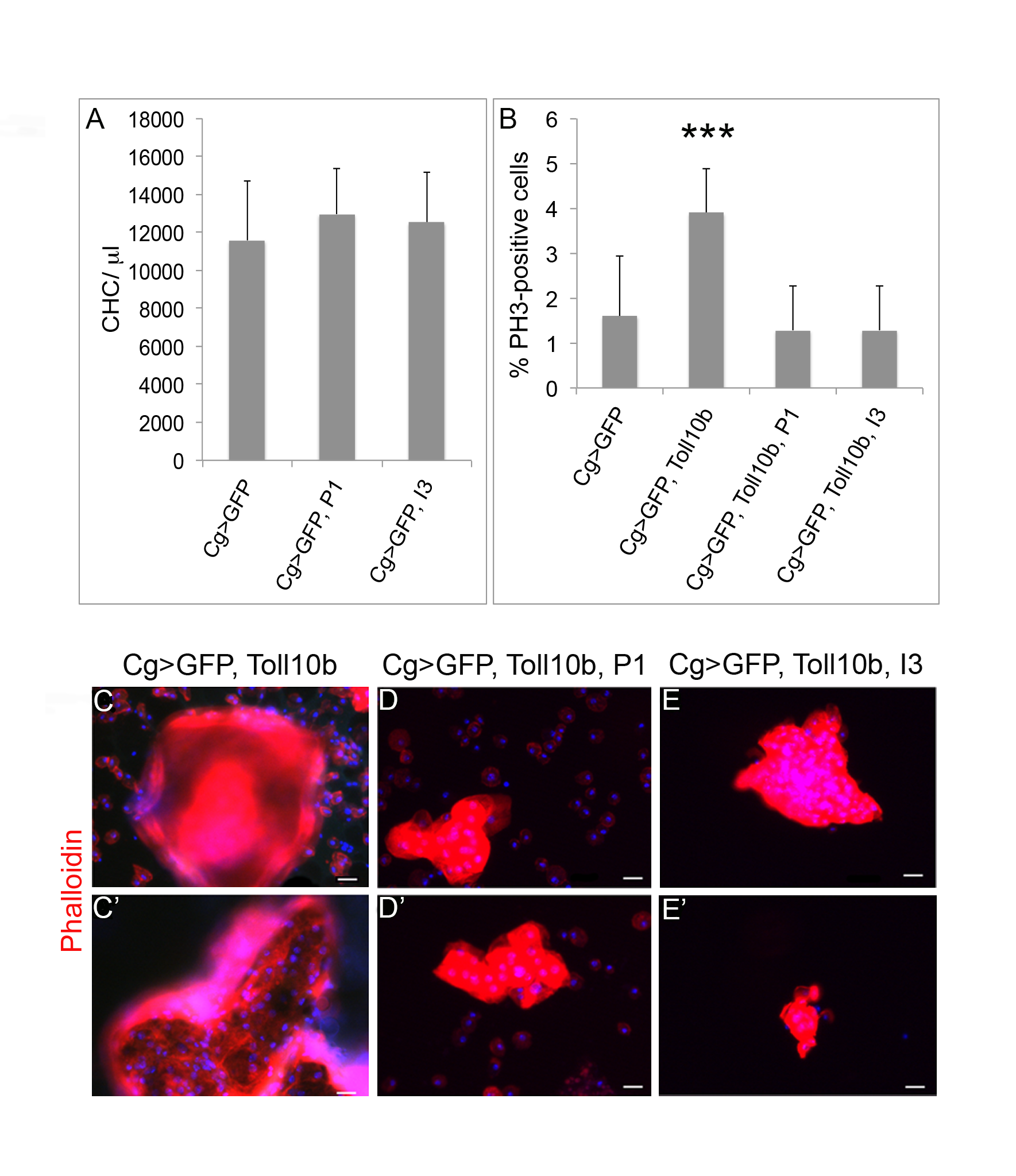

Supplement: Figure S3 — Effects of Vankyrins on circulating blood cell population, and on mitosis and tumors due to hyperactive Toll activity. A. Cg>GFP driven Vankyrin (2 copies each of P1 or I3) expression does not significantly affect circulating hemocyte concentration (CHC). In all cases, CHC is within the control range [7]. Comparison between control Cg>GFP and Cg>GFP, P1 larvae: (11,585±3,131 cells versus 12,966±2,374 cells; t = −1.4, df = 27.96, p = 0.17. N = 16) and Cg>GFP versus Cg>GFP, I3 larvae: (11,585±3,131 cells versus 12,539±2,612 cells; t = −0.9, df = 29.07, p = 0.36. N = 16). All cells in all three genotypes are GFP-positive. B. Expression of Cg>Toll10b increases the proportion of phospho-histone H3 (PH3)-positive cells (Wilcoxon test W = 10.5, p<0.01. N = 8 for Cg>GFP; N = 14 for Cg>GFP, Toll10b), while Vankyrin expression reverses this effect (Wilcoxon test W = 4.5, p<0.01 for Cg>Toll10b, GFP compared to Cg>Toll10b, GFP, P1; Wilcoxon test W = 7.5, p<0.01 for Cg>Toll10b, GFP compared to Cg>Toll10b, GFP, I3. N = 14 for Cg>Toll10b, GFP; N = 11 for Cg>Toll10b, GFP, P1 and N = 27 for Cg>Toll10b, GFP, I3). Vankyrin expression reverses mitosis to control levels (Wilcoxon test W = 42.5, p = 0.86 for Cg>GFP compared to Cg>Toll10b, GFP, P1 and Wilcoxon test W = 94.5, p = 0.61 Cg>GFP compared to Cg>Toll10b, GFP, I3. N = 8 for Cg>GFP; N = 11 for Cg>Toll10b, GFP, P1 and N = 27 for Cg>Toll10b, GFP, I3). C–E′. Melanized tumors from C–C′ Cg>GFP, Toll10b, D–D′ Cg>GFP, Toll10b P1, or E–E′ Cg>GFP, Toll10b I3 larvae. (TIF) [file ppat.1003580.s003.tif]
