# Supplementary material for: Polydnaviral Ankyrin Proteins Aid Parasitic Wasp Survival by Coordinate and Selective Inhibition of Hematopoietic and Immune NF-kappa B Signaling in Insect Hosts
Source: PLoS Pathog. 2013 Aug 29;9(8):e1003580. doi: 10.1371/journal.ppat.1003580 (PMC3757122; doi:10.1371/journal.ppat.1003580)
Supplement: Text S1 — Nucleotide sequences of primers used for polymerase chain reaction amplification conditions (number of amplification cycles and melting temperatures) used for different genes considered in this study. (DOC) [file ppat.1003580.s004.doc]

Supplementary Text 1. PCR primers and amplification conditions.

| **Target** | **Primers** | **PCR conditions** | **References** |
| --- | --- | --- | --- |
| I3 | CsIV-I3. CCGGAATTCCGAAAATGGATTACAAGGACGACGATGACGATAAAGAAAATTCTCAAATTGCAAAG | 30 cycles, Tm = 40°C | This study |
|  | pUAST-R-Xba1. CTATCTAGATCATCAATGCTGATTGTCATTCGAT |  |  |
| P1 | CsIV-P1. CCG GAA TTC CGA AAA TGG ATT ACA AGG ACG ATG ACG ATA AA G AGA TTT CTC AAA TTC GAA AG | 30 cycles, Tm = 40°C | This study |
|  | pUAST-R-Xba1. CTATCTAGATCATCAATGCTGATTGTCATTCGAT |  |  |
| Drosomycin | Dro-F. ATCCTGAAGTGCTGGTGCGAAGGA | 40 cycles, Tm = 59°C | 2 |
|  | Dro-R. ACGTTCATGCTAATTGCTCATGG |  |  |
| ProPO59 | PO59-F. GAGAGCATCCAGGTGGAGAG | 40 cycles, Tm = 59°C | This study |
|  | PO59-R. TAGCTGAATTCGTGGTGCTG |  |  |
| ProPO54 | PO54-F. CAACTGGCTTCGTTGAGTGA | 40 cycles, Tm = 59°C | This study |
|  | PO54-R. CGGGCAGTTCCAATACAGTT |  |  |
| ProPO45 | PO45-F. AAGATGCGGGAGGAGTCTTT | 40 cycles, Tm = 59°C | This study |
|  | PO45-R. TCGAAGGGGTAGACCAGATG |  |  |
| Rp49 (reference gene) | Rp-F. GACGCTTCAAGGGACAGTATCTG | 40 cycles, Tm = 59°C | 2 |
|  | Rp-R. AAACGCGGTTCTGCATGAG |  |  |
